# Land cover changes across Greenland dominated by a doubling of vegetation in three decades

**DOI:** 10.1038/s41598-024-52124-1

**Published:** 2024-02-13

**Authors:** Michael Grimes, Jonathan L. Carrivick, Mark W. Smith, Alexis J. Comber

**Affiliations:** School of Geography and Water@Leeds, University of Leeds, Woodhouse Lane, Leeds, LS2 9JT UK

**Keywords:** Climate sciences, Climate change, Environmental sciences, Environmental impact, Geomorphology, Cryospheric science

## Abstract

Land cover responses to climate change must be quantified for understanding Arctic climate, managing Arctic water resources, maintaining the health and livelihoods of Arctic societies and for sustainable economic development. This need is especially pressing in Greenland, where climate changes are amongst the most pronounced of anywhere in the Arctic. Ice loss from the Greenland Ice Sheet and from glaciers and ice caps has increased since the 1980s and consequently the proglacial parts of Greenland have expanded rapidly. Here we determine proglacial land cover changes at 30 m spatial resolution across Greenland during the last three decades. Besides the vastly decreased ice cover (− 28,707 km^2^ ± 9767 km^2^), we find a doubling in total areal coverage of vegetation (111% ± 13%), a quadrupling in wetlands coverage (380% ± 29%), increased meltwater (15% ± 15%), decreased bare bedrock (− 16% ± 4%) and increased coverage of fine unconsolidated sediment (4% ± 13%). We identify that land cover change is strongly associated with the difference in the number of positive degree days, especially above 6 °C between the 1980s and the present day. Contrastingly, absolute temperature increase has a negligible association with land cover change. We explain that these land cover changes represent local rapid and intense geomorphological activity that has profound consequences for land surface albedo, greenhouse gas emissions, landscape stability and sediment delivery, and biogeochemical processes.

## Introduction

The Arctic has been warming at double the global mean rate since the 1970s^1^. Some of the most pronounced recent warming has been across Greenland, where mean annual air temperatures between 2007 and 2012 were 3 °C warmer compared to the 1979–2000 average^2^. More extremes of temperature and precipitation are expected in the near future as Greenland’s climate resilience decreases and non-linear land-climate system feedbacks develop^3^, including soil development and vegetation change, land surface albedo change, and permafrost degradation. The environmental impacts of Arctic climate change are most obviously manifest in Greenland’s abundant, expanding and rapidly-evolving proglacial landscapes^4^. Specifically, Greenland’s glaciers and ice caps (GICs) are shrinking, glacier-fed lakes are expanding, permafrost lakes are draining, rivers are transporting vast amounts of sediment and aggrading and widening, and vegetation cover and species diversity is expanding, largely coincident with Arctic shrubification^5–12^.

Understanding ongoing climate-landscape interactions across Greenland is crucial for modelling Arctic climate^13^, monitoring and managing water resources^14^, determining the health and livelihoods of Arctic societies^15^, and for maximising economic development prospects^16^. Specifically in Greenland, climate-landscape feedbacks include: (i) exposure of dark surfaces such as water and bedrock that have a high absorption of solar energy, i.e. a low albedo) causing increased thermal retention^17,18^; (ii) increased deposition of organic matter, both terrestrially and within streams and eventually fjords as a consequence of warming-induced soil formation and permafrost degradation which drives further colonization of vegetation in areas where sediment redistribution is common and stream networks diverge regularly^19^; (iii) expedited degradation of permafrost by vegetation establishment and expansion subsequently releasing substantial previously stored greenhouse gases^12,20,21^; and (iv) increased/altered biogeochemical weathering and reactions coinciding with changes in microbial community composition driven by warming and increased moisture within the environment which affects gas (e.g. CO_2_, O_2_, N_2_O) production and sequestration rates^22,23^. The rates of change associated with these feedbacks is highly uncertain, not least because quantification of changing land cover in Greenland is needed to identify sensitive sites and to elucidate the driving earth surface processes.

Large-scale analyses of land cover changes have been hindered by a lack of computational power and the relative scarcity of satellite imagery prior to 2000. Previous analyses of land cover change across Greenland have either been spatially-localised (e.g.^24–26^), limited to modern twenty-first century changes (e.g.^27,28^), or specialist in scope regarding the specific type of land cover observed (e.g. Jørgensen et al.’s^29^ study of vegetation in north east Greenland, Heindel et al.’s^25^ study of soil erosion in Kangerlussuaq). When regional analyses have been undertaken for Greenland they have been relatively coarse (> 1 km) resolution (e.g.^30,31^), which precludes analysis of important intra-catchment or ‘process-form system’ dynamics (cf.^32^), or not specifically designed for and verified in Greenland; e.g. CALC-2020 land cover map of the Arctic using multi-band Sentinel imagery^33^. There has been no quantification of, or accounting for, the complex land cover responses across Greenland to accelerated atmospheric warming since the late 1980s.

Here we report spatiotemporal land cover change between the late 1980s and 2010s across Greenland. We achieve this land cover change detection at 30 m spatial resolution, thereby permitting analysis and understanding of both inter- and intra-catchment earth surface processes that drive and explain regional land cover change patterns. We present a novel land cover phase change conceptual model for Greenland based on our spatially-aggregated measurements of predominant inter-class changes, and we apply a rigorous spatially-distributed assessment of landcover change accuracy directly. Our datasets have widespread application within the geosciences and more widely in land management and natural resource-based economic sectors.

### Classifying land cover and quantifying land cover changes

**Figure 1:**
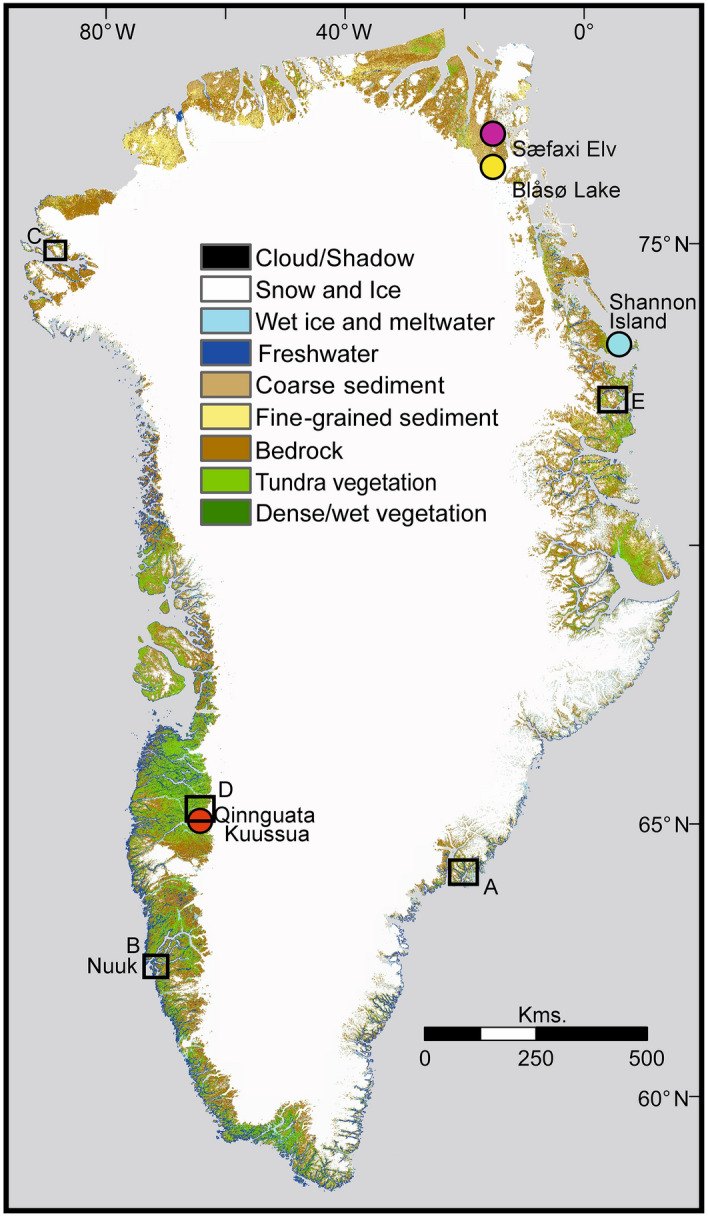
Landsat-8-derived land cover classification for the 2010s. The coloured circles and site names indicate where we highlight analysis of catchment-scale processes in Fig. 3, and the black squares with letters denote five locations within which are our 89 field validation points detailed in Fig. SI.6.

We designed and implemented a rigorous pre-processing and classification workflow, as fully detailed in our Supplementary Information (SI), leveraging Google Earth Engine (GEE) computing power to analyse imagery from two time periods; Landsat-5 Thematic Mapper (TM) for the late 1980s (July to Sept, 1986 to 1989 inclusive) and Landsat-8 Operational Land Imager (OLI) for the late 2010s (July to Sept, 2016 to 2019 inclusive). Misclassification from differing illumination conditions due to topographic shading and differing solar positions during image acquisition was reduced by applying a topographic correction^34^ and a pixel quality weighted mosaicking routine. The latter produced a mosaic from summer month images on a pixel-by-pixel basis using a weighted median filter based on the Near InfraRed band to reduce the inclusion of low reflectance (shadow) and over-saturated (snow, cloud edge). We implemented a semi-supervised Random Forest classification^35^ to derive nine broad land cover classes; bad data (NoData/cloud/shadow), ice and snow, meltwater (e.g. rivers and sediment plumes), freshwater, coarse sediment, fine sediment, bedrock, dry tundra, and wet/dense vegetation (Fig. 1). Detailed class definitions are given in our SI. These nine classes were chosen to represent the major land cover of the biophysical environment whilst reducing redundancy (too many classes) and maintaining accuracy (distinction between classes). The overall accuracy, which we derive using a proportional area error estimation method^36^ (SI), is 85% for our 1980s classification and 82% for our2010s classification. The full methodology and results of accuracy assessment, adjustment for error, and calculation of confidence intervals is outlined in the accompanying Supplementary Information.

To directly address the accuracy of changes detected we constructed a 64 class change image and correspondence matrix, for which we found change accuracy of 69%. We identified that misclassification predominantly occurs between spectrally-similar classes and error is spatially heterogeneous, so we aggregated classes with similar geophysical and geomorphological characteristics and computed spatial distribution of error via geographically weighted binomial regression (SI).

To identify spatial patterns, percentage area coverage of each of the nine broad land cover classes were spatially-aggregated on a 10 × 10 km^2^ grid for each time period (SI). To understand drivers of the regional pattern of land cover changes, and due to sparse and intermittent field measurements of weather across Greenland (e.g.^37^), we compared our observed large-scale patterns of land cover change to Copernicus Climate Change Service (C3S) ERA5 modelled air temperature datasets^38^. Intra-catchment processes were detected and analysed by exploiting the high spatial resolution (30 m) of our land cover classifications to identify exemplar sites of land cover change types.

### Regional patterns of land cover change

**Figure 2:**
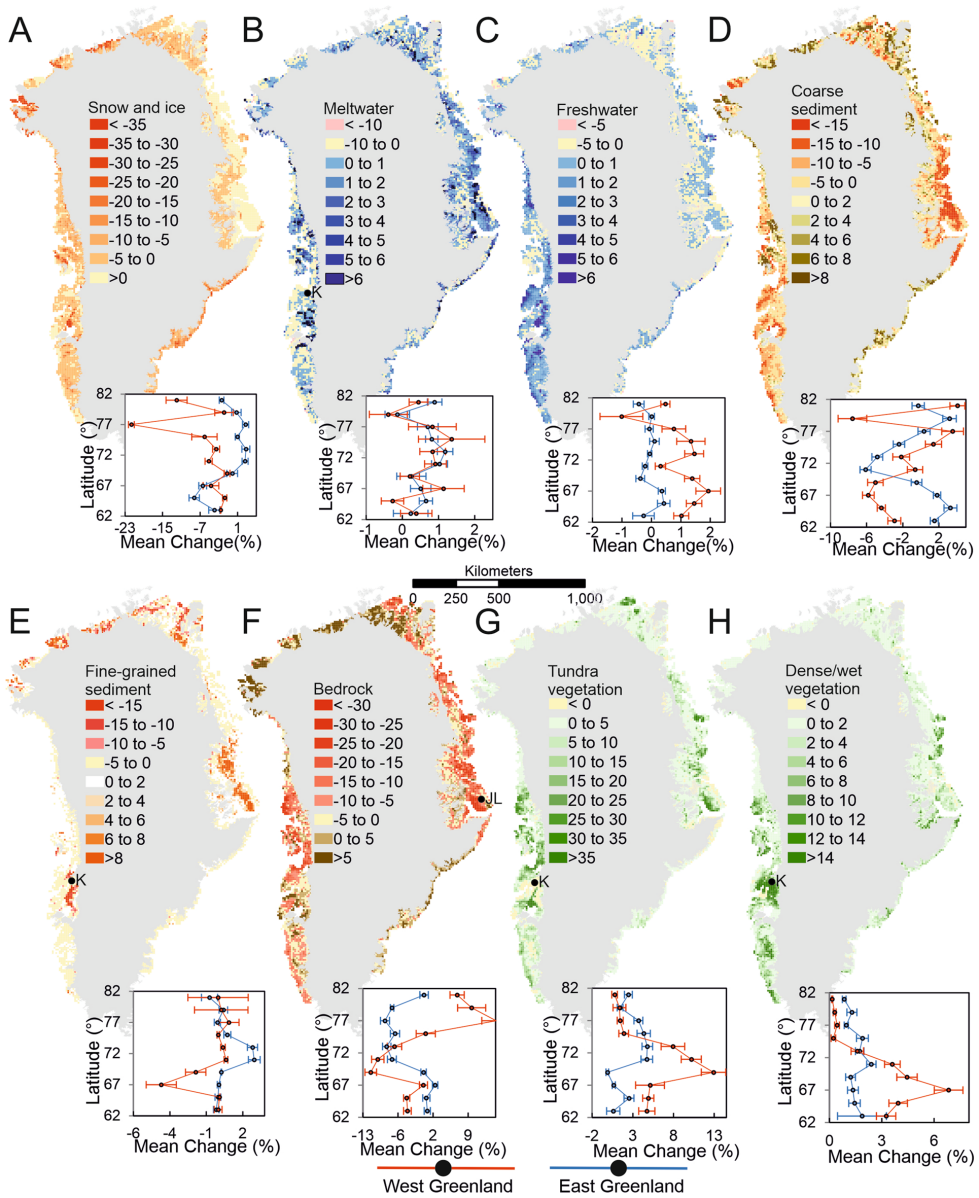
Percentage of total area change within 10 km × 10 km grid cells for each land cover class and plots of variation in average land-cover change with latitude for both east and west Greenland and 95% Confidence intervals. Inset *K* Kangerlussuaq, *JL* Jameson Land.

Overall, we find there has been a 28,707 km^2^ ± 9767 km^2^ areal loss of snow/ice from the ice sheet margin and GICs, and that has predominantly been replaced by barren ground; bedrock and coarse sediment. The losses of ice volume associated with these land cover changes represent considerable contributions to global sea level rise and ocean freshening^39^ and will also control river discharge and associated sediment plumes in fjords affecting coastal ecosystems (e.g.^40^). As expected, we detect ice loss concentrated around the edges of present-day glacier margins, but we also here reveal that it has been especially pronounced in the north and the south-west of Greenland (Fig. 2A) (c.f.^41^). Highest ice losses of − 22% (> 22 km^2^ of a single 10 × 10 km^2^ cell) are found around 77° N in the west, with relatively high melt rates being observed in the mid-north–west (~ − 6% areas, 70° N to 74° N) and South-east (~ − 8% areas, 62° N to 67° N) (Fig. 2A). Our geographically-weighted binomial regression analysis indicated that change from snow/ice accuracy was lower in the north west than other regions, although still averaging over 80% (SI). These patterns of ice cover loss are corroborated by Mouginot et al.^42^ who reported glacier mass balance from the 1972 to 2018, finding particularly high increases in ice flux (almost 100%) and cumulative loss (~ 150 Gt) from basins in mid/north-west Greenland. Ice loss is the most marked land cover change for albedo, which is a key component of surface energy balance and hence a control on micro-climate(s).

Changes in meltwater and freshwater classes have been spatially-heterogeneous, showing a slight positive correlation with latitude on both the east and west coast (Fig. 2B), and a negative trend for freshwater ponds and lakes (Fig. 2C). Across Greenland, there has been a 15% ± 15 (10,031 km^2^ ± 10,516 km^2^) increase in meltwater area, which we interpret to represent increased to river discharge and large increases in meltwater-sediment plumes in lakes and shallow parts of fjords. Our meltwater changes (Fig. 2B) are corroborated by findings from Mernild et al.^43^, who found increasing runoff trends for 80% of Greenland’s catchments between 1979 and 2014 with relatively high runoff in the south-west and north-east, as we identify in Fig. 2B. This increased meltwater river discharge impacts both local fjord/coastal waters in terms of sea surface temperature, salinity, suspended sediment and stratification, all of which influence marine ecosystems^43^. More widely, meltwater increase and consequent mobilisation of fluvial sediment can increase ocean freshening and impact Atlantic meridional overturning circulation^44^ besides impacting the delicate specialist marine ecosystems of the Arctic^45,46^.

Perhaps unexpectedly, we find a decrease in freshwater area of 11% ± 22% (− 4336 km^2^ ± 8421 km^2^). However, that decreased area of freshwater is due to degrading permafrost and some episodic fluxes, such as from glacier lake drainages; for example. Finger-Higgens et al.^47^ reported a 2% decrease in lake area (15% for smaller ponds) in west Greenland, and Carrivick and Quincey^48^ and How et al.^49^ both show changes in lakes adjacent to ice-margins.

Barren ground classes have undergone incoherent spatial changes (Fig. 2D, E, F). Notable areas with loss of bedrock and coarse/dark sediment occur in the mid/north-east and mid-west of Greenland (Fig. 2D, F) and predominantly in ice-distal locations. The net change of coarse sediment is negligible at − 0.5% ± 6% (− 1183 km^2^ ± 15,455 km^2^) but there has been a − 16% ± 4% (67,553 km^2^ ± 16,682 km^2^) loss in exposed bedrock. We attribute the latter to vegetation encroachment and growth on weathered bedrock regolith surfaces and largely via the process of shrubification due to warming temperatures^50^, as studied on Disko Island, Greenland^51^, for example, and more widely quantified herein. Indeed, losses in the sediment and bedrock classes coincide spatially with increases in both vegetation classes; dry tundra and dense/wet vegetation (Fig. 2G) and these sites are also found inside the zone of highest permafrost thaw potential^21^. Exposure time since deglaciation (and prior to 1980) in these locations is sufficient (~ 10,000 years^52^) for soil and rudimentary peat development to be represented in our 1980s sediment and bedrock classes, producing optimal sites for vegetation encroachment during this study period^53^. Overall, loss of barren ground represents the stabilisation of land surfaces and consequently decreased sediment fluxes, which ultimately affects mineral and nutrient export to the oceans^54,55^.

Fine sediment coverage has not significantly but likely increased overall across Greenland; 4% ± 13% (4274 km^2^ ± 13,239 km^2^), but spatially corresponds with increases in meltwater (Fig. 2B and E). Fine sediment movement represents increasing sediment mobility and unstable land surfaces and is commonplace during deglaciation (cf.^55^) and with thawing and drying of land. Fine sediment loss has particularly occurred in south-west Greenland in the vicinity of Kangerlussuaq ~ 67° N (Fig. 2E). In the same area, there has been an increase in exposure of bedrock and a negligible change in dry tundra vegetation, which is otherwise uncharacteristic in the south-west (Fig. 2E, F, G). These land cover changes can be explained by expansion of deflation patches, which are proceeding at rates of ~ 2.5 cm yr^−1^^56^. There is also a spatial association in south-west Greenland between this aeolian erosion of fine sediment (sand and silt) and the increases in cover of vegetation. On the east coast of Greenland, fine-grained sediment has increased at 72 ^o^N in Jameson Land and that corresponds with reduced bedrock coverage and increased coverage of meltwater with ice-loss further inland. The exposed bedrock in Jameson Land is an extensive Late Jurassic sandstone complex^57^ and increased slope instability from permafrost degradation combined with increased glacial meltwater action and deposition has likely caused the marked increase in fine-grained sediment^58^.

Both vegetation classes have in combination increased in coverage across Greenland by 111% ± 12% (87,475 km^2^ ± 15,288 km^2^). We find pronounced increases in vegetation cover across the south-west, east, and north-east (Fig. 2G). The greatest increase in coverage of dense/wet vegetation has occurred in the vicinity of Kangerlussuaq in the south-west and isolated patches in the north-east (Fig. 2H). Most of this new dense/wet vegetation coverage can be attributed to wet heath development along receding lake shores between 1995 and 2017^59^. Jørgensen et al.^29^ identify similar patterns of fenland distribution in the north-east (~ 2012) as those in our contemporary classification, but studies from the mid-late twentieth century^60–62^ indicated far less extensive dense and wetland vegetation across the north-east, as depicted in Fig. 2H. Overall, changes in both vegetation cover classes show a latitudinal gradient; a pattern of increasing change with latitude to between ~ 63 ^o^N and 69 ^o^N and decreased change north of this (Fig. 2G, H). Vegetation changes are especially important because they represent a stabilisation of land, introduce seasonal albedo variability and strongly affect snow retention, infiltration and overall local boundary layer (micro)climate. Moreover, vegetation development stabilises hillslopes reducing sediment and nutrient delivery to watercourses, yet simultaneously expedites permafrost degradation and introduces carbon and dissolved organic carbon and other greenhouse gases to the terrestrial^63^, fluvial^64^, oceanic^65^ and atmospheric environment^66^.

### Geomorphological processes

**Figure 3:**
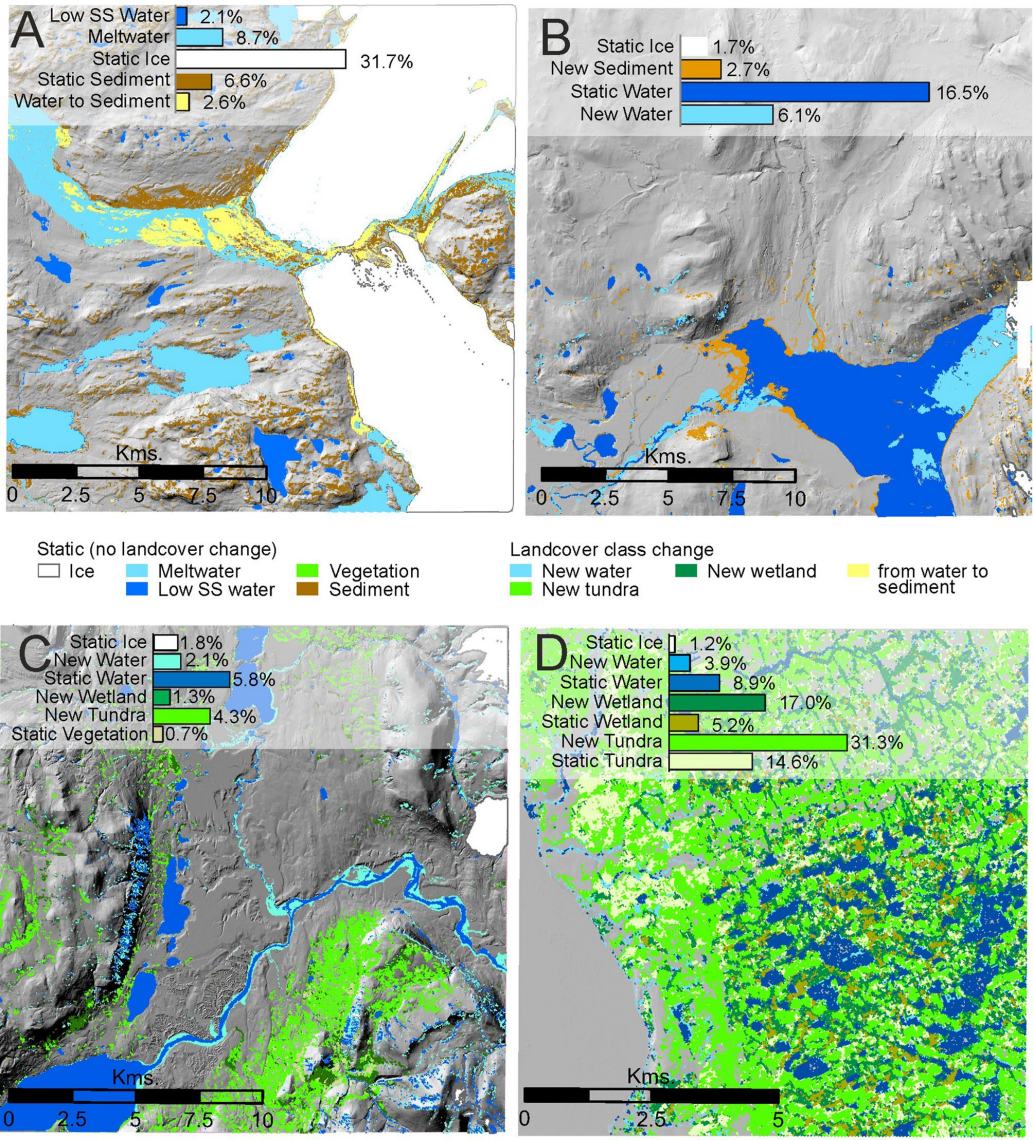
Examples of localised land cover change demonstrating geomorphological processes driving environmental evolution as seen using changes in 30 m resolution land cover data. (**A)** Proglacial sedimentation (Qinnguata Kuussua river), (**B)** delta progradation (Blåsø lake), (**C)** increased meltwater stream size and discharge (Sæfaxi Elv), and (**D**). vegetation and wetland growth (Shannon Island). SS = Suspended Sediment. Locations for sites in panels are shown in Fig. 1.

Four locations (see Fig. 1 for locations) can be chosen to exemplify landscaping processes and resultant landform and land cover changes that are representative of the spatial trends and that are permitted by the native 30 m resolution of our analysis. Figure 3A shows the southernmost source of the Qinnguata Kuussua river in south-west Greenland (Fig. 2E) where meltwater is primarily subglacially-fed and the absence of glacier surface drainage reflects low precipitation and relatively little supraglacial melt^67,68^. The proglacial river braidplain is complex in planform and composition (Fig. 3A) but is notable for the expansion in coverage of 3% or ~ 10 km^2^ of the 400 km^2^ selected area of fine-grained sediment, reflecting aggradation of fluvially-transported sediment. This type of braidplain expansion and altered river morphology resulting from sediment aggradation has far reaching implications. Terrestrial sediment deposition and anastomising may reflect reduced energy and so reduced nutrient conveyance marine ecosystems^69^. However, as sand reserves are depleted globally and demand increases (e.g. urban expansion, infrastructural improvements, coastal protection), Greenland may be poised to exploit this widespread phenomenon (4% increase in fine sediment across Greenland) to become a leading global aggregate exporter for sustainable economic independence^70^.

Figure 3B evidences increased sediment flux and consequent river changes around the ice-dammed lake Blåsø in north-east Greenland. There have been river channel avulsions and sedimentation across 3% or ~ 11 km^2^ of the 400 km^2^ area and that has most obviously occurred as delta progradation. New sediment is only found around the mouths of meltwater streams (Fig. 3B), thereby refuting attribution to a falling lake level. Increased meltwater discharge, which is ubiquitous around Greenland (Fig. 2B) and the associated fluvial mobilisation of sediment is most likely to be causing the delta progradation. Where sediment supply is abundant, i.e. regions we identify as increasing in sediment and bare ground classes largely in proglacial landscapes (Fig. 2D, E, F), the meltwater rivers transport that sediment ultimately driving widespread progradation in lakes and fjords (Fig. 3B). These geomorphological processes are important because deltas considerably alter coastal and lacustrine morphodynamics, sediment delivery ratios and connectivity with knock-on implications for ecosystems dependent on nutrients present in glacigenic sediment (e.g.^45,71–73^).

Figure 3C shows the Sæfaxi Elv river which drains Centrum Lake eastward to the ocean via Hekla Sound in King Frederick VIII Land, north-east Greenland. There has been a 4% or 16 km^2^ increase in the surface area of the river over the 400 km^2^ area, which can be predominantly attributed to the Greenland-wide increase in meltwater discharge^7,43^; we find meltwater river area increases across Greenland of 15% coverage, though a large proportion of this will encompass sediment plumes into lakes and fjords, as well as some wet ice. We also note considerable increase in both vegetation classes in this area (Fig. 3C), and evidences the spatial association of vegetation encroachment and growth with increased availability of freshwater sourced from melting GICs and degrading permafrost^74^.

We find a near quadrupling (increase of 380% ± 29%, 30,295 km^2^ ± 6805 km^2^) in the coverage of wet/dense vegetation across Greenland. Wetland expansion on Shannon Island, east Greenland (Fig. 3D) is an exemplar of widespread wetland development and expansion in east and north-east Greenland (Fig. 2H). Jørgensen et al.^29^ identified wetlands and fen in this region and proposed that the wetlands would continue to expand with permafrost degradation. These wetlands are likely minerotrophic, principally deriving nutrients from glacial meltwater, and constitute considerable sources of CH_4_ in polar regions^75^. Locations of wetland formation and growth in north-east Greenland identified here present key sites for research to explore the poorly understood process of wetland, and peat, initiation and lateral expansion, a field at the forefront of ongoing arctic biospheric research^76,77^. Arctic wetland development has a complex and poorly understood biogeochemical connotation for greenhouse gas emission versus drawdown; Arctic wetlands are effective methane emitters^78^, yet dry and moist arctic tundra soils in north east Greenland have been measured to draw down methane^29^. Expansion of vegetation and especially in wetland areas indicates but also exacerbates permafrost thaw, active layer thickening and thus emissions of greenhouse gases previously stored in these Arctic soils^79^.

### Association of land cover change with climate change

We conducted a number of spatial regression analyses to determine how climate change is associated with the land cover changes. The difference in the average (per decade) number of days per year above various significant positive degree temperature thresholds was more informative than absolute magnitude of temperature change because of the confounding influences of temperature and precipitation. Consider a three degree warming that remains below zero versus a three degree warming that crosses from below to above zero thereby leading to ice melt and permafrost thaw, for example. Comparing our land cover change grids to our Difference in Degree Days Above Temperature grids (between 1980 and 2010s) on a grid cell by grid cell basis, we found lowest *R*^2^ and Akaike information criterion values (SI) for the two water and the fine sediment classes. We find the highest *R*^2^ and Akaike information criterion correlations for the vegetation, bedrock and ice classes, suggesting a close association between these types of land cover change and climate change across Greenland. Specifically and most interestingly, the highest *R*^2^ and Akaike information criterion values for all classes are found for the difference in the number of days above 6 °C. This leads us to suggest that future land cover change is therefore likely to be marked and accelerated in regions where the number of days per year above this 6 °C threshold are increasing most rapidly; crucially that is not simply where temperatures are set to rise most rapidly. Table SI 12 contains all geographically weighted regression results.

### A model of changing land cover across Greenland

**Figure 4:**
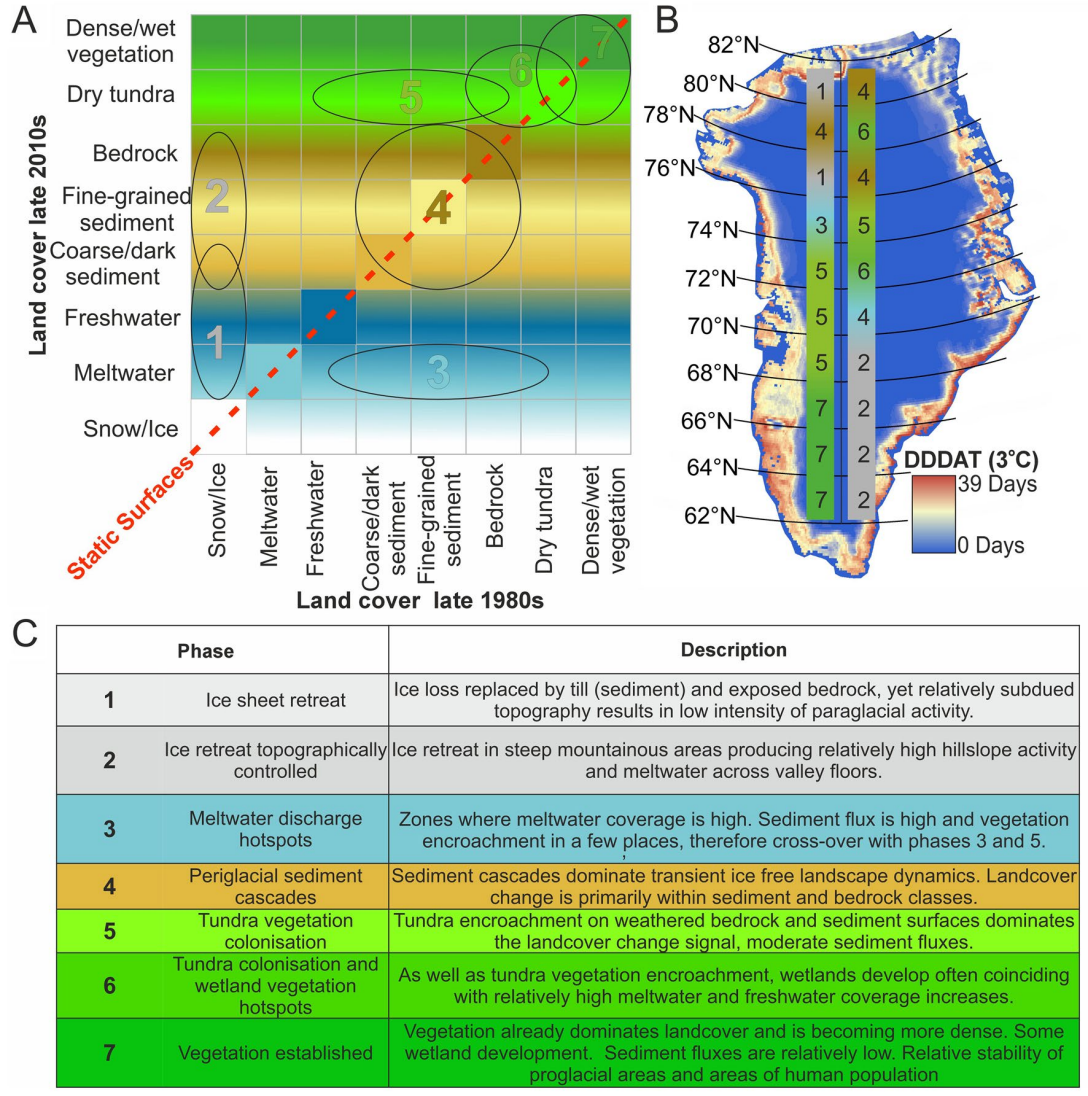
Land cover change model 1980s to 2010s for Greenland, illustrating numbered predominant landcover inter-class changes on a common trajectory reflecting increasing landscape stability with time; i.e. phases (**A**). Panel (**B)** shows degree day difference air temperature (DDDAT) mapped and with east and west coast latitudinal transects of numbered predominant landcover inter-class change in each latitudinal band. Numbered inter-class changes are detailed in the table (**C**).

We summarise both regional inter-class changes and intra-catchment geomorphological processes that we have measured (Figs. 2, 4A) within a conceptual model (Fig. 4A, B) that is based upon 64 class change matrices. Each of those 64 class changes is a calculation of every ‘from-to’ change type between classes (Fig. 4C), and considering both relative change within each matrix as well as relative change per class between matrices (SI). We highlight that there is a notable difference in the latitudinal pattern of the predominant land cover inter-class changes per latitudinal band between the east and west of Greenland (Figs. 2, 4B). The size of proglacial areas is far larger on the west coast than in the east, and the topographic relief is generally more subdued in the west compared to the alpine east and so accommodating of regolith and tundra vegetation establishment. Moreover, the two coasts have experienced different climatic change^37^, in terms of temperature and precipitation; the east is influenced by variability in North Atlantic Oscillation and the Atlantic Meridional Overturning Circulation.

Our model, and the relationship between land cover changes and the difference in degree days above 6 °C, illustrates that higher air temperatures are producing more suitable conditions for vegetation expansion and growth^50^. Vegetation cover is perhaps the most profound land cover change we have quantified because it has a considerable feedback effect on the climate system^80^. For example, the newly-established and enlarging wetlands and fens in phase 6 regions of the north-east of Greenland are associated with considerable methanogenesis^81^, but that is potentially partially offset by long-term carbon storage via peat accretion^82^. Field observations combined with mapping indicate widespread shrubification in Greenland, whereby the “greening” of the Arctic is largely manifest in the proliferation of shrub tundra species^83^.

We identify substantial vegetation areal expansion and growth in regions of critical permafrost thaw (phases 5 and 6, Fig. 4). That expansion can be interpreted as a manifestation of increased air temperature warming ground surfaces and driving permafrost thaw^84^. Permafrost thaw impacts a suite of human and economic activity; Greenland’s infrastructure has been forced onto challenging ground underlain by thick poorly consolidated glacigenic sediment and permafrost^21^ with economic development, localised population growth and increased tourism that all have environmental consequences (c.f.^85^). Our analysis can therefore be interpreted to reveal a positive feedback between permafrost degradation and vegetation encroachment that will likely increase exponentially, threating both local communities and infrastructure^15^. These land cover feedbacks, especially for landscape parts in phases 5 through 7 (Fig. 4), therefore warrant policymakers’ attention.

Our study has identified, quantified and summarised regional, inter-catchment and intra-catchment land cover changes across Greenland between the late 1980s and the late 2010s. This spatiotemporal coverage has enabled proposal of a conceptual model of land cover phase change and the association of that land cover change with climate. Specifically, we find that where the difference in degree days is > 6 °C, then we find increased ice-sheet and glacier mass loss, increased meltwater production and increased sediment mobility. Overall, the rapid changes in land cover across Greenland requires repeated monitoring to identify change trajectories and to refine understanding, which will directly benefit human and economic development. For the geosciences, feedbacks of land cover with climate must be considered when modelling and projecting climate, the Greenland Ice Sheet, GIC evolution, meltwater runoff, sediment yield and habitat and ecological community changes.

## Methods

Here we briefly outline the main methods for producing our image mosaics and classification. Further details can be found in our Supplementary information (SI).

### Image preparation

Landsat-8 Operational Land Imager (OLI) Top of Atmosphere (TOA) imagery is used for the contemporary classification, and Landsat-5 Thematic Mapper TM TOA imagery is used for the late 1980s classification. Landsat imagery is the most widely used data type for land cover mapping and classification largely due to it having the longest satellite image record spanning nearly 50 years^86^, it being freely available, and its relatively high 30 m resolution^87–89^. TOA products were chosen over atmospherically corrected surface reflectance (SR) as the US Geological Survey (USGS)^90^ state the efficacy of surface reflectance correction is reduced in: (i) highly snow-covered regions, (ii) regions with low-sun angles, (iii) coastal regions where land area is small relative to adjacent ocean, (iv) areas with high cloud cover conditions, and (v) high latitude areas over 65° North^90^. As Greenland’s peripheral ice-free areas meet most/all of these criteria, SR products should not be used for analysis. Summer month images (July–September) are selected for the years 1986 to 1989 inclusive for the late eighties and 2016–2019 inclusive for the contemporary mosaic. We produced multi-month multi-year mosaics as: (i) the temporal resolution is limited by the 16-day return period for Landsat imaging, (ii) cloud cover and orographic shadowing is particularly problematic in certain regions of Greenland^91^, and (iii) there is poor Landsat coverage for the south, south west, and north west of Greenland during the late eighties^92^. Full workflow is outlined in (Figure SI.1).

### Image classification

In the first instance, the classification was conducted using an unsupervised, k-means clustering algorithm executed in GEE. This method removes the users potential to target predefined purpose-driven classes which may be difficult to identify or that are presumed to be in abundance when relatively spatially sparse^93,94^. The unsupervised approach is particularly useful for large, national/regional scale spatial analysis where it is unreasonable for the user to cover the entire area for validation and therefore only classes which can be well distinguished and are spectrally distinct are produced. The initial classification workflow is presented in Figure SI.4. Once a classification is produced with K number of classes, these are then aggregated into 9 land cover classes. These final 9 land cover classes were chosen as they are deemed distinct and identifiable from the imagery, present across the entire periphery of Greenland, relevant to this research and enough as to reduce the production of redundant classes. These classes and their descriptions are shown in Table SI. 1.

### Classification accuracy assessment

Accuracy of the contemporary classification was assessed using four band (RGB,NIR) 3 m resolution contemporary PlanetScope basemap mosaic imagery from the third quarter of 2019 (July–September) as reference^95^ and a Sentinel-SA image mosaic for 2019 summer season produced in Google Earth Engine. Due to a lack of supplementary satellite imagery for the 1980s, our Landsat mosaic was used for reference alongside 2 m resolution greyscale georeferenced aerial orthophotographs, made available by the AeroDEM project^96^. We report an overall accuracy for our classifications to one significant figure only; 85% for our 1980s classification and 82% for the 2010s classification. Below we outline in detail our accuracy assessment procedure using the contemporary classification. This same method was also applied to our eighties classification.

Accuracy assessment was conducted for six 100 km × 100 km areas south of 76° N shown in Figure SI.2. PlanetScope imagery is not available north of 76° N. Stratified random sampling based on total occurrence of classes for the entirety of Greenland was used to generate 500 accuracy assessment points (AAP) distributed between the 8 land cover classes at each location. Results were then amalgamated resulting in 3000 AAP over total area of 60,000 km^2^. We removed the ice sheet from analysis as this skewed the stratified AAP production and is largely static over the study period away from the margins. Snow and ice accuracy was therefore assessed using GICs. A correspondence (AKA: error, confusion) matrix was produced using the 3000 AAP and is presented both absolutely and as an expression of proportional area considering the associated error within each class, following Olofsson et al.^36^. The overall kappa coefficient is 0.781, and overall accuracy (OA) was presented as 82%. The basic kappa coefficient however adjusts for ‘random allocation agreement’ and the validity of the adjustment has been questioned^98,99^. We therefore also used a stratified estimation of error following Olofsson et al.^36^ to mitigate against potential inherent measurement bias and to estimate standardised errors with quantified uncertainty based on sampling variability. The cell-count derived correspondence matrix is presented in Table SI.2.

### Field validation

In order to further validate our contemporary classification we collected field validation at a spatially distributed array of 89 sites as mapped and detailed in Figure SI.6. Photographs of each land cover type are presented in Figure SI.7. We found our field validation points to have an overall kappa coefficient of 0.81, in line with the accuracy we reported from remote validation.

### Classification change accuracy assessment

To confirm that the accuracy of the individual classifications is reflected in the changes we measure, we directly quantify the accuracy of changes. Using Google Earth Engine (GEE) a 64 class change map for the entirety of Greenland was produced (available as supplementary dataset). See Table SI.5 for legend describing the 64 change class designations.

A further 3000 AAP were randomly generated, stratified by the occurrence of each of the 64 classes in Google Earth Engine. The minimum number of points for each class was set at 5, as some classes has especially low areas which equated to less than 1 point when stratified by relative areas (e.g. class 56: Dense vegetation to Snow/ice). The accuracy of these changes was then assessed using the same reference data as was used for individual class accuracy assessment, i.e. Sentinel-2A mosaic and Planet imagery for contemporary, Landsat mosaic and 2 m AeroDEM orthophotographs for the 1980s. The overall Kappa statistic of accuracy for the change image correspondence matrix 0.69. Whilst conducting change class accuracy assessment it was evident that accuracy was not spatially homogenous, and misclassifications were predominantly between classes with largely similar spectral signatures and associated geomorphological process attributions. We therefore explored the spatial distribution of error following class aggregation as outlined in Table SI.6. The specifics and findings of spatially distributed error estimation are outlined in our SI.

### Associations with climate

We conducted a number of regression analyses to understand the degree to which Greenland’s climate change has influenced the land cover changes we observe over the thirty year study period. Decadal and annual average summer temperature change from the European Centre for Medium-Range Weather Forecasts (ECMWF) ERA-5 monthly average dataset for summer months was correlated with the change classes, but Ordinary Least Squared (OLS) correlations were all below 0.1 and indicated a non-parametric relationship. We speculated that average increase in temperature is not a useful an indicator of land cover in Greenland because changes above 0 °C will produce ice melt and permafrost thawing, whereas a change (of equal magnitude) below zero will not. We therefore calculated the difference in the average number of days in a year above three significant positive degrees temperature thresholds (0 °C, 3 °C and 6 °C) for the late 1980s and late 2010s which we term Difference in Degree Days Above Temperature (DDDAT) grids, produced from ECMWF ERA5 land-hourly gridded data and prepared in Google Earth Engine.

We averaged the number of days per year where temperatures were above the three threshold values (0 °C, 3 °C, and 6 °C) for the years 1986, 1987, 1988, and 1989 and took the average of these four years to have an average Degree Days Above Threshold (DDAT) value for each threshold temperature for the late eighties. We then did the same for the years 2016, 2017, 2018, and 2019 for the contemporary DDAT value. Differencing these grids then gave us the Difference in Degree Days Above Temperature (DDDAT) grid. Zonal statistics was then used to aggregate these values to the 10 km × 10 km fishnet. Climate data is spatially non-stationary, and nearby sites in space tend to have similar values of temperature/temperature change over time than would be expected by chance. This inherent spatial autocorrelation in the data makes the application of classical linear and parametric tests such as OLS problematic due to the innate assumption of independently distributed error^100^.

In the OLS model, the variables to be related are assumed to the spatially stationary and that at each point of the study area the globally quantified relationship is constant and that the model is absolutely representative. When comparing environmental variables such as land cover change and their climatic driving variables this has been shown to rarely be the case (Propastin et al., 2008). Geographically Weighted Regression (GWR) regresses locally and allows the parameters to vary across space as a moving kernel with a set bandwidth or neighbourhood. It is incorrect to hold that for land cover types with variable responses to climate across a diverse and large spatial area a single linear model is applicable at every point^101–104^.

### Ethics declarations

The authors affirm that all research conducted for this study adheres to the highest ethical standards. The research presented does not involve human subjects, human communities, or any data related to human individuals. All methodologies employed were carried out with respect to the environment and the landscapes studied, ensuring minimal harm and disturbance.

### Supplementary Information

Supplementary Information.

## Data Availability

The datasets used and/or analysed during the current study are made available from the corresponding author upon on reasonable request. There are no restrictions on data availability.

## References

[1] Chylek, P. et al. Arctic air temperature change amplification and the Atlantic Multidecadal Oscillation. *Geophys. Res. Lett*. **36** (2009).

[2] Mayewski PA (2014). Holocene warming marked by abrupt onset of longer summers and reduced storm frequency around Greenland. J. Quat. Sci..

[3] Overland JE (2020). Less climatic resilience in the Arctic. Weather Clim. Extremes.

[4] Carrivick JL, Heckmann T (2017). Short-term geomorphological evolution of proglacial systems. Geomorphology.

[5] Anderson NJ (2017). The Arctic in the twenty-first century: Changing biogeochemical linkages across a paraglacial landscape of Greenland. BioScience.

[6] Overeem I (2017). Substantial export of suspended sediment to the global oceans from glacial erosion in Greenland. Nat. Geosci..

[7] Bamber JL, Westaway RM, Marzeion B, Wouters B (2018). The land ice contribution to sea level during the satellite era. Environ. Res. Lett..

[8] Box JE (2018). Global sea-level contribution from Arctic land ice: 1971–2017. Environ. Res. Lett..

[9] Box JE (2019). Key indicators of Arctic climate change: 1971–2017. Environ. Res. Lett..

[10] Teufel B, Sushama L (2019). Abrupt changes across the Arctic permafrost region endanger northern development. Nat. Clim. Change.

[11] Shugar DH (2020). Rapid worldwide growth of glacial lakes since 1990. Nat. Clim. Change.

[12] Mekonnen ZA (2021). Arctic tundra shrubification: A review of mechanisms and impacts on ecosystem carbon balance. Environ. Res. Lett..

[13] Hibbard K (2010). Research priorities in land use and land-cover change for the Earth system and integrated assessment modelling. Int. J. Climatol..

[14] Pekel JF (2016). High-resolution mapping of global surface water and its long-term changes. Nature.

[15] Hjort J (2018). Degrading permafrost puts Arctic infrastructure at risk by mid-century. Nat. Commun..

[16] Ford JD, Goldhar C (2012). Climate change vulnerability and adaptation in resource dependent communities: A case study from West Greenland. Clim. Res..

[17] Lunt DJ, de Noblet-Ducoudre N, Charbit S (2004). Effects of a melted Greenland ice sheet on climate, vegetation, and the cryosphere. Clim. Dyn..

[18] Mernild, S.H. et al. Detailed spatiotemporal albedo observations at Greenland's Mittivakkat Gletscher. *EGUGA*, **2643** (2015).

[19] Lindborg T (2016). Biogeochemical data from terrestrial and aquatic ecosystems in a periglacial catchment, West Greenland. Earth Syst. Sci. Data.

[20] Yi S, Woo MK, Arain MA (2007). Impacts of peat and vegetation on permafrost degradation under climate warming. Geophys. Res. Lett..

[21] Daanen RP (2011). Permafrost degradation risk zone assessment using simulation models. The Cryosphere.

[22] Barcena TG, Finster KW, Yde JC (2011). Spatial patterns of soil development, methane oxidation, and methanotrophic diversity along a receding glacier forefield, Southeast Greenland. Arct. Antarc. Alp. Res..

[23] Musilova M (2017). Microbially driven export of labile organic carbon from the Greenland ice sheet. Nat. Geosci..

[24] Elberling B (2008). Soil and plant community-characteristics and dynamics at Zackenberg. Adv. Ecol. Res..

[25] Heindel RC, Chipman JW, Virginia RA (2015). The spatial distribution and ecological impacts of Aeolian soil erosion in Kangerlussuaq, West Greenland. Ann. Assoc. Am. Geogr..

[26] Palmtag J (2018). Landform partitioning and estimates of deep storage of soil organic matter in Zackenberg, Greenland. The Cryosphere.

[27] Raynolds MK (2019). A raster version of the circumpolar arctic vegetation map (CAVM). Remote Sens. Environ..

[28] Karami M (2018). A phenology-based approach to the classification of Arctic tundra ecosystems in Greenland. ISPRS J. Photogramm. Remote Sens..

[29] Jørgensen CJ (2015). Net regional methane sink in High Arctic soils of northeast Greenland. Nat. Geosci..

[30] Loveland TR (2000). Development of a global land-cover characteristics database and IGBP DISCover from 1 km AVHRR data. Int. J. Remote Sens..

[31] Walker D, Gould W, Maier H, Raynolds M (2002). The circumpolar arctic vegetation map: AVHRR-derived base maps, environmental controls, and integrated mapping procedures. Int. J. Remote Sens..

[32] MacMillan RA, Shary PA (2009). Landforms and landform elements in geomorphometry. Dev. Soil Sci..

[33] Liu C (2023). CALC-2020: A new baseline land cover map at 10 m resolution for the circumpolar Arctic. Earth Syst. Sci. Data.

[34] Soenen SA, Peddle DR, Coburn CA (2005). SCS+ C: A modified sun-canopy-sensor topographic correction in forested terrain. IEEE Trans. Geosci. Remote Sens..

[35] Breiman L (2001). Random forests. Mach. Learn..

[36] Olofsson P (2013). Making better use of accuracy data in land change studies: Estimating accuracy and area and quantifying uncertainty using stratified estimation. Remote Sens. Environ..

[37] Box JE (2002). Survey of Greenland instrumental temperature records: 1873–2001. Int. J. Climatol..

[38] Hersbach H (2020). The ERA5 global reanalysis. Q. J. R. Meteorol. Soc..

[39] Rignot E (2011). Acceleration of the contribution of the Greenland and Antarctic ice sheets to sea level rise. Geophys. Res. Lett..

[40] Rysgaard S, Nielsen TG, Hansen BW (1999). Seasonal variation in nutrients, pelagic primary production and grazing in a high-Arctic coastal marine ecosystem, Young Sound, Northeast Greenland. Mar. Ecol. Prog. Ser..

[41] Shepherd A (2020). Mass balance of the Greenland Ice Sheet from 1992 to 2018. Nature.

[42] Mouginot J (2019). Forty-six years of Greenland ice sheet mass balance from 1972 to 2018. Proc. Natl. Acad. Sci..

[43] Mernild, S.H. et al. Reconstruction of the Greenland ice sheet surface mass balance and the spatiotemporal distribution of freshwater runoff from Greenland to surrounding seas. *The Cryosphere Discuss.,* **1–50** (2017).

[44] Sejr MK (2017). Evidence of local and regional freshening of Northeast Greenland coastal waters. Sci. Rep..

[45] Hawkings JR (2015). The effect of warming climate on nutrient and solute export from the Greenland Ice Sheet. Geochem. Perspect. Lett..

[46] Oliver H (2018). Exploring the potential impact of Greenland meltwater on stratification, photosynthetically active radiation, and primary production in the Labrador Sea. J. Geophys. Res. Oceans.

[47] Finger Higgens RA (2019). Changing lake dynamics indicate a drier Arctic in Western Greenland. J. Geophys. Res. Biogeosci..

[48] Carrivick JL, Quincey DJ (2014). Progressive increase in number and volume of ice-marginal lakes on the western margin of the Greenland Ice Sheet. Glob. Planet. Change.

[49] How P (2021). Greenland-wide inventory of ice marginal lakes using a multi-method approach. Sci. Rep..

[50] Myers-Smith IH (2011). Shrub expansion in tundra ecosystems: dynamics, impacts and research priorities. Environ. Res. Lett..

[51] Callaghan TV, Christensen TR, Jantze EJ (2011). Plant and vegetation dynamics on Disko Island, West Greenland: Snapshots separated by over 40 years. Ambio.

[52] Bennike O, Björck S (2002). Chronology of the last recession of the Greenland ice sheet. J. Quat. Sci..

[53] Romanovsky VE, Smith SL, Christiansen HH (2010). Permafrost thermal state in the polar Northern Hemisphere during the international polar year 2007–2009: A synthesis. Permafr. Periglac. Process..

[54] Moreau M (2008). Impacts of recent paraglacial dynamics on plant colonization: A case study on Midtre Lovénbreen foreland, Spitsbergen (79 N). Geomorphology.

[55] Carrivick JL, Tweed FS (2021). Deglaciation controls on sediment yield: Towards capturing spatio-temporal variability. Earth-Sci. Rev..

[56] Heindel RC, Culler LE, Virginia RA (2017). Rates and processes of Aeolian soil erosion in West Greenland. The Holocene.

[57] Surlyk F, Noe-Nygaard N (2001). Sand remobilisation and intrusion in the upper Jurassic Hareelv formation of East Greenland. Bull. Geol. Soc. Den..

[58] Gruber S, Haeberli W (2007). Permafrost in steep bedrock slopes and its temperature-related destabilization following climate change. J. Geo-phys. Res. Earth Surf..

[59] Law AC, Nobajas A, Sangonzalo R (2018). Heterogeneous changes in the surface area of lakes in the Kangerlussuaq area of southwestern Greenland between 1995 and 2017. Arct. Antarct. Alp. Res..

[60] Winther PC (1950). A preliminary account of the Danish Pearyland expedition, 1948–9. Arctic.

[61] Bay, C. Review of vegetation mapping in Greenland. In *Circumpolar Arctic Vegetation Mapping Workshop* (1994).

[62] Jakobsen BH (1992). Preliminary studies of soils in North-East Greenland between 74 and 75 northern latitude. Geogr. Tidsskr. Dan. J. Geogr..

[63] Horwath Burnham J, Sletten RS (2010). Spatial distribution of soil organic carbon in northwest Greenland and underestimates of high Arctic carbon stores. Glob. Biogeochem. Cycles.

[64] Pastor A (2021). Geomorphology and vegetation drive hydrochemistry changes in two Northeast Greenland streams. Hydrol. Process..

[65] Brovkin V (2002). Carbon cycle, vegetation, and climate dynamics in the Holocene: Experiments with the CLIMBER-2 model. Glob. Bio-geochem. Cycles.

[66] Ström L (2003). The effect of vascular plants on carbon turnover and methane emissions from a tundra wetland. Glob. Change Biol..

[67] van As D (2011). Surface mass budget and meltwater discharge from the Kangerlussuaq sector of the Greenland ice sheet during record-warm year 2010. Cryosphere Discuss..

[68] Ryu JS, Jacobson AD (2012). CO_2_ evasion from the Greenland ice sheet: A new carbon-climate feedback. Chem. Geol..

[69] Makaske B (2001). Anastomosing rivers: A review of their classification, origin and sedimentary products. Earth-Sci. Rev..

[70] Bendixen M (2019). Promises and perils of sand exploitation in Greenland. Nat. Sustain..

[71] Bendixen M (2017). Delta progradation in Greenland driven by increasing glacial mass loss. Nature.

[72] Hendry KR (2019). The biogeochemical impact of glacial meltwater from Southwest Greenland. Prog. Oceanogr..

[73] Martin JB (2020). Comparisons of nutrients exported from Greenlandic glacial and deglaciated watersheds. Glob. Biogeochem. Cycles.

[74] Rasmussen LH (2018). Modelling present and future permafrost thermal regimes in Northeast Greenland. Cold Reg. Sci. Technol..

[75] Huttunen JT (2003). Methane emissions from natural peatlands in the northern boreal zone in Finland, Fennoscandia. Atmos. Environ..

[76] Walvoord MA, Kurylyk BL (2016). Hydrologic impacts of thawing permafrost—A review. Vadose Zone J..

[77] Kreplin HN (2021). Arctic wetland system dynamics under climate warming. Wiley Interdiscip. Rev. Water.

[78] O'Connor FM (2010). Possible role of wetlands, permafrost, and methane hydrates in the methane cycle under future climate change: A review. Rev. Geophys..

[79] Hollesen J, Elberling B, Jansson PE (2011). Future active layer dynamics and carbon dioxide production from thawing permafrost layers in Northeast Greenland. Glob. Change Biol..

[80] Normand S (2013). A greener Greenland? Climatic potential and long-term constraints on future expansions of trees and shrubs. Philos. Trans. R. Soc. B.

[81] Kotsyurbenko, O.R. et al. Methanogenesis in soils, wetlands, and peat. *Biogenesis of Hydrocarbons, Handbook of Hydrocarbon and Lipid Microbiol*, **1–18** (2019).

[82] Ovenden L (1990). Peat accumulation in northern wetlands. Quat. Res..

[83] Myers-Smith IH (2020). Complexity revealed in the greening of the Arctic. Nat. Clim. Change.

[84] Schuur EA (2015). Climate change and the permafrost carbon feedback. Nature.

[85] Arnaut J, Lidman J (2021). Environmental sustainability and economic growth in Greenland: Testing the environmental Kuznets curve. Sustainability.

[86] Joshi PK, Ghosh A, Chakraborty A, Sharma R, Joshi A (2013). Landsat again—continuing remote sensing, monitoring, mapping and measuring. Curr. Sci..

[87] Cohen WB, Goward SN (2004). Landsat’s role in ecological applications of remote sensing. BioScience.

[88] Tucker CJ, Grant DM, Dykstra JD (2004). NASA’s global orthorectified landsat data set. Photogramm. Eng. Remote Sens..

[89] Wulder MA (2008). Landsat continuity: Issues and opportunities for land cover monitoring. Remote Sens. Environ..

[90] Landsat missions. Landsat Missions | U.S. Geological Survey Available at: https://www.usgs.gov/landsat-missions. (Accessed 20 Jun 2021)

[91] Marshall G, Dowdeswell J, Rees W (1994). The spatial and temporal effect of cloud cover on the acquisition of high quality landsat imagery in the European arctic sector. Remote Sens. Environ..

[92] Goward S (2006). Historical record of landsat global coverage. Photogramm. Eng. Remote Sens..

[93] Tømmervik H (2003). Monitoring vegetation changes in Pasvik (Norway) and Pechenga in Kola Peninsula (Russia) using multitemporal Landsat MSS/TM data. Remote Sens. Environ..

[94] Hasmadi M, Pakhriazad HZ, Shahrin MF (2009). Evaluating supervised and unsupervised techniques for land cover mapping using remote sensing data. Geogr. Malays. J. Soc. Space.

[95] Planet Team. Planet Lab (2017). Available at: https://www.planet.com/ (Accessed: 01/06/2022).

[96] Korsgaard, N. J. et al. Digital elevation model and orthophotographs of Greenland based on aerial photographs from 1978–1987. *Sci. Data* **3** (2016). 10.1038/sdata.2016.32 PMC4862325 27164457

[97] Foody GM (2002). Status of land cover classification accuracy assessment. Remote Sens. Environ..

[98] Pontius RG, Millones M (2011). Death to kappa: Birth of quantity disagreement and allocation disagreement for accuracy assessment. Int. J. Remote Sens..

[99] Clifford P, Richardson S, Hemon D (1989). Assessing the significance of the correlation between two spatial processes. Biometrics.

[100] Wang J, Price KP, Rich PM (2001). Spatial patterns of NDVI in response to precipitation and temperature in the Central Great Plains. Int. J. Remote Sens..

[101] Propastin P, Kappas M, Erasmi S (2008). Application of geographically weighted regression to investigate the impact of scale on prediction uncertainty by modelling relationship between vegetation and climate. Int. J. Spat. Data Infrastruct. Res.

[102] Li S, Zhao Z, Miaomiao X, Wang Y (2010). Investigating spatial non-stationary and scale-dependent relationships between urban surface temperature and environmental factors using geographically weighted regression. Environ. Model. Softw..

[103] Zhou H, Wang J, Wan J, Jia H (2009). Resilience to natural hazards: A geographic perspective. Nat. Hazards.

[104] Zhao N, Yang Y, Zhou X (2010). Application of geographically weighted regression in estimating the effect of climate and site conditions on vegetation distribution in Haihe catchment, China. Plant Ecol. China.

