# Supplementary material for: Land cover changes across Greenland dominated by a doubling of vegetation in three decades
Source: Sci Rep. 2024 Feb 13;14:3120. doi: 10.1038/s41598-024-52124-1 (PMC10864356; doi:10.1038/s41598-024-52124-1)
Supplement: Supplementary file 1 — Supplementary Information. [file 41598_2024_52124_MOESM1_ESM.docx]

Supporting information for

**Land cover changes across Greenland dominated by a doubling of vegetation in three decades**

Michael Grimes^1^, Jonathan L. Carrivick^1^, Mark W. Smith^1^, Alexis Comber^1^

^1^School of Geography and water@leeds, University of Leeds, Woodhouse Lane, Leeds, LS2 9JT. UK.

**Contents of this file**

- Text and diagrams outlining Landsat image mosaic and classification process
- Text on classification accuracy assessment including equations and error matrixes
- Text on production of regional patterns of landcover change maps
- Text on method of local scale process driving change analysis
- Text on Difference in Degree Days Above Temperature (DDDAT) grid production and geographically weighted regression method with tables of geographically weighted regression results per class.
- Text on transition model production and latitudinal to-from class matrix method with all matrices included.
- Tables SI.1 to SI.12
- Figures SI.1 to SI.14

**Additional Supporting Information (Files/Links Uploaded separately)**

- **Data Set S1.** Our contemporary (2016, 2017, 2018, 2019) and 1980s (1986, 1987, 1988, 1989) Landsat best pixel mosaic images are made freely available and are presented in Google Earth Engine with script to export to google drive.
- **Data Set S2.** Our 1980s and contemporary landcover classifications are made freely available and are presented in Google Earth Engine with script to export to google drive.

1. **Image preparation**

Rigorous pre-processing of Landsat imagery is required in order to produce the most accurate and highest quality image mosaic representing landcover at each point on the surface at the respective time periods, and to negate many of the limitations imposed on imagery from Greenland. We developed and implemented a rigorous Landsat image pre-processing procedure (Figure SI. 1). We leverage and utilise the parallel processing power of Google Earth Engine (GEE) to conduct this pre-processing for the first time at a national scale and 30m resolution in Greenland. GEE is a cloud based computing environment with access to a wide variety of open source datasets, including the full archive of Landsat imagery (Gorelick et al., 2017). The large parallel computing platform enables high-resolution analysis over various spatial and temporal timescales. The entire respective Landsat collections are filtered temporally, spatially, and based on the cloud score before being topographically corrected using the Modified Sun-Canopy-Sensor method (cf. Soenen et al., 2005).


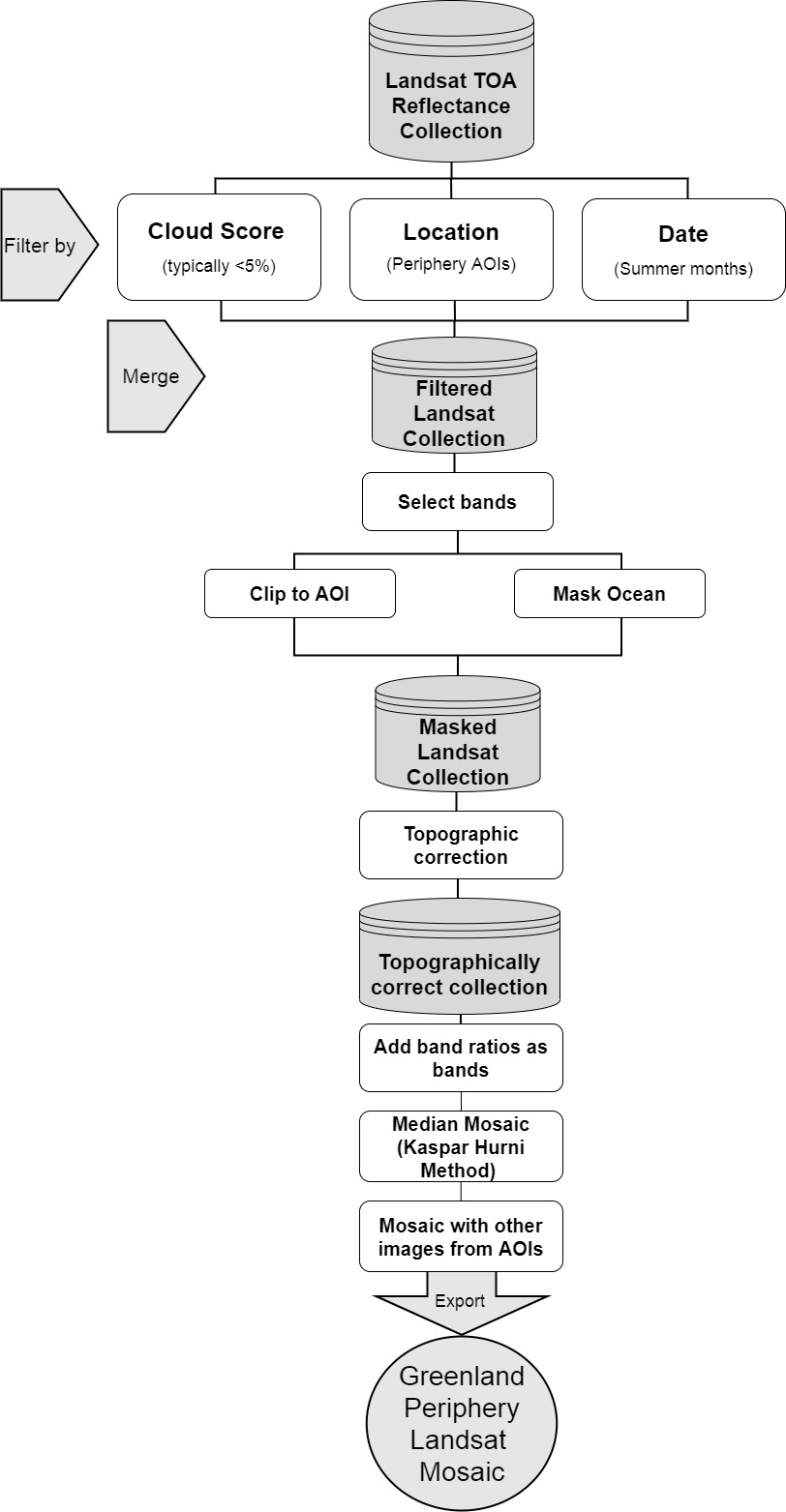


**Figure SI.1**. Pre-processing workflow implemented in Google Earth Engine (GEE) for Landsat imagery

The first stage is to filter the entire Landsat-5 (1980s) and Landsat-8 (contemporary) TOA collections by date so that only summer months are considered due to lower snow cover and higher sun angles to reduce shadows. Then the collection is further filtered by area, so only tiles which overlap an area of interest (AOI) are considered (Fig. SI.2) Of these tiles, only images with low cloud cover over land (typically <5 %) are selected, with this information extracted from images metadata. The dimensionality of this final multi-year merged collections is reduced by only selecting 6 bands (Blue, Green, Red, NIR, SWIR1, SWIR2) in each image and creating a new image collection. The image collection is then run through a clipping function whereby each image in the collection is clipped to the area of interest (Figure SI.2). The Greenland Ice Mapping Project (GIMP) created a mask of oceans and ice through manual digitisation from Landsat -7 imagery from around the year 2000 (Howat et al., 2014). The GIMP ocean mask data is used in a masking function, which masks all ocean pixels from each image in the clipped image collection. Some manual adjustments to the masks are required as the GIMP masks are created from images collected in around the year 2000 and some noise/gaps exist.


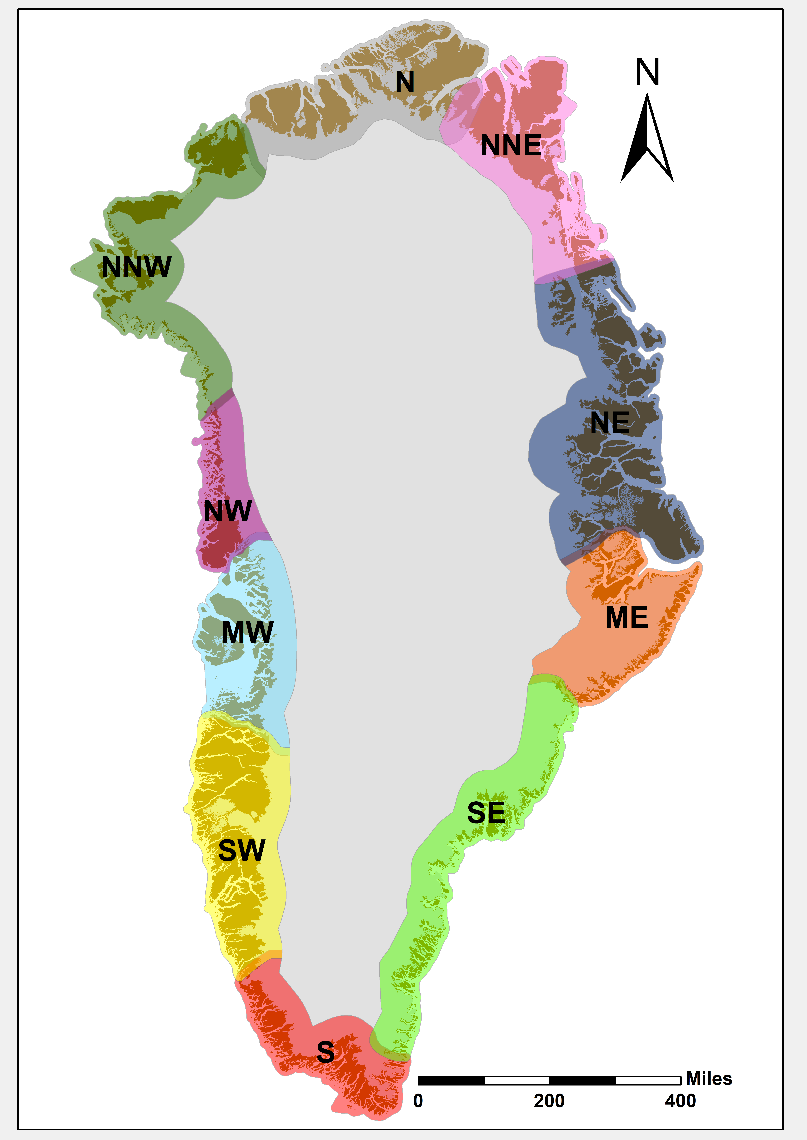


**Figure SI.2**. Areas of interest (AOI) referenced in the text. N = North, NNE = North-North East, NE = North East, ME = Mid-East, SE = South East, S = South, SW = South West, MW = Mid-West, NW = North West, NNW = North-North West. Black spots represent locations where accuracy assessment was conducted (Section 3)

Topographic correction is the process by which topographic effects are accounted for by alteration of cell band values based on satellite metadata (solar zenith and azimuth angles) and a digital elevation model from which aspect and slope are calculated. Topographic correction is often more important than atmospheric correction in topographically complex and high latitude regions, vastly improving the accuracy of landcover classification in these areas (Vanonckelen et al., 2013). The Modified Sun-Canopy-Sensor Topographic Correction method, as outlined in (Soenen et al., 2005) was applied to all images within the collection to account for topographic effects. Essentially, the method uses the sun-canopy-sensor (SCS) with a semi-empirical moderator to account for diffuse radiation. Topographic corrections utilised the GIMP DEM, due to its complete spatial coverage of Greenland and its 30m resolution matching the resolution of the Landsat imagery. The source code for the topographic correction method was found in Poortinga et al. (2019), however that was written for use on surface reflectance imagery with precise solar zenith and azimuth angles in image metadata. The code was therefore adapted here for use with TOA Landsat data through adjustments to the function for illumination condition (IC) calculation and image metadata about solar position. Due to the high computational cost, with the topographic correction algorithms applied to every cell of every band in every image within the collection, even in GEE computations timed out and memory allocations were exceeded for large areas, therefore the periphery was dissected into smaller areas with overlap. Figure SI.3 shows an example of an area in the far northeast of Greenland in the Sermersooq Municipality before and after topographic correction.


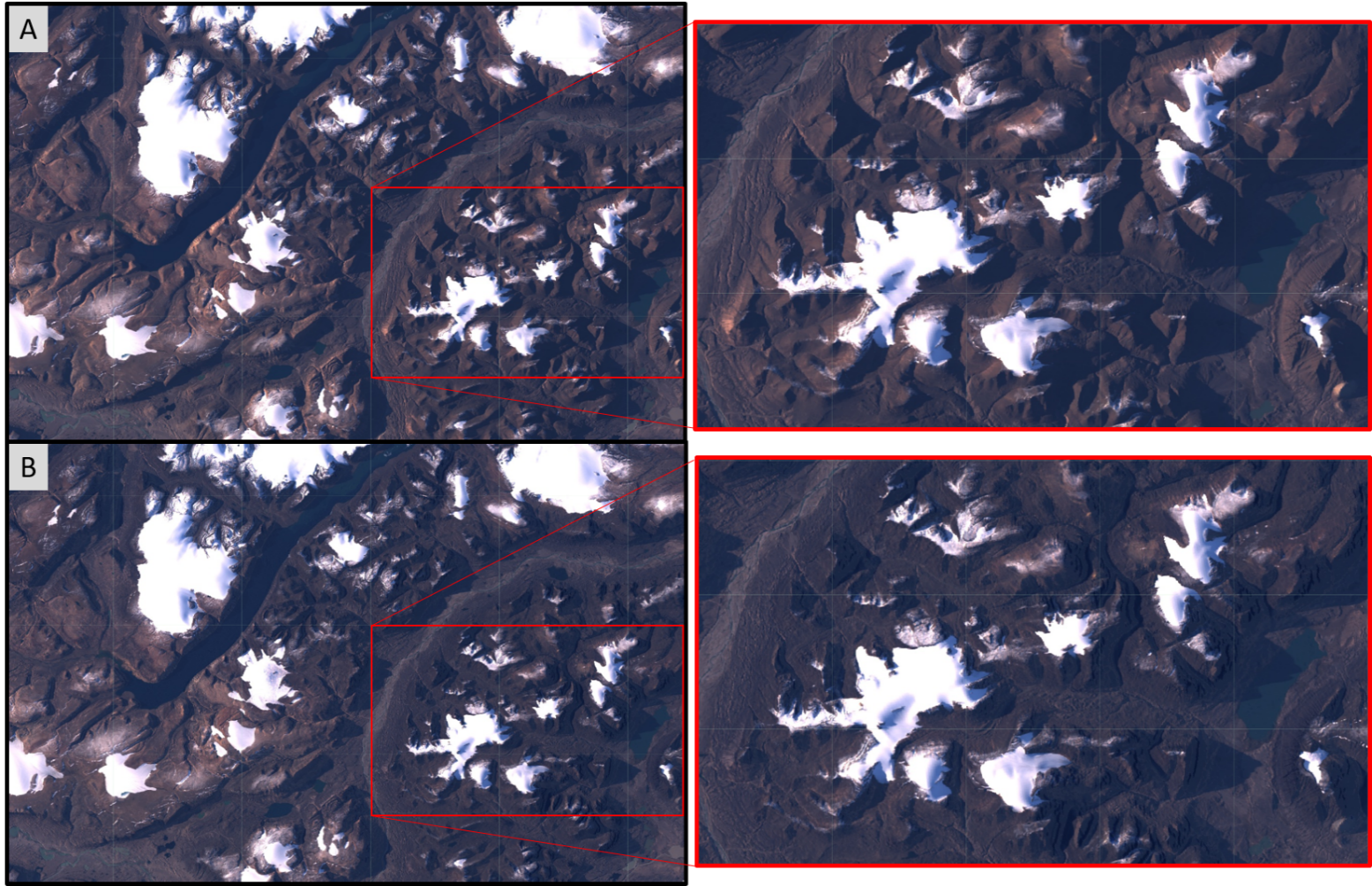


**Figure SI.3.** Example of pre (A) and post (B) true-colour Landsat 8 imagery (Northeast Greenland, Sermersooq Municipality). Green inset boxes highlight the removal of differences in illumination on slopes ‘in pre’ image (A), and how this “flattened” and removed in the ‘post’ image(B). As the illumination condition on the hillslopes is corrected, the same weathered surfaces are spectrally indistinct in the ‘post’ image (B), where before it appeared lighter when the hillslope aspect faced the sun’s position and may incorrectly have been classified as a separate land cover by the classification algorithm.

Following topographic correction, a function to compute and add band ratios as bands is applied to the images in the topographically corrected collection. Band ratios were included due to their enhancement of spectral differences between bands to highlight specific landcover types, as well as to further negate the impacts of topography, aspect and shadowing (Kloiber et al., 2002). The normalized difference vegetation index (NDVI) is a well-established ratio indicator of vegetation cover (Tucker, 1979; Bannari et al., 1995). As well as effectively differentiating vegetated from non-vegetated areas, NDVI has been shown to highlight vegetation properties including leaf area, fractional vegetation cover, vegetation condition and biomass (Carlson and Ripley, 1997). The equation for the NDVI is given in equation (1).

|  | $NDVI= \frac{NIR-Red}{NIR+Red}$ | (1) |
| --- | --- | --- |

The normalized difference snow index (NDSI) finds use in discriminating snow/ice from snow and ice-free areas (Kulkarni et al., 2002; Nolin, 2010). Though high reflectance of snow and ice often leads to saturation in the visible bands of the Landsat sensors, including green (B3) used in the NDSI, this shortcoming is most pronounced at lower latitudes where incoming solar radiation is highest and resultantly so too are levels of reflected radiation over comparable surfaces (Selkowitz and Forster, 2015). The NDSI also allows for improved differentiation between snow and ice variants such as fresh/dry snow, wet snow, wet ice and bare ice (Hall and Riggs, 2011). The equation for the NDSI is given in equation (2).

|  | $NDSI= \frac{Green-SWIR}{Green+SWIR}$ | (2) |
| --- | --- | --- |

|  | $NDWI= \frac{Green-NIR}{Green+NIR}$ | (3) |
| --- | --- | --- |

The NDWI is also included as a means to effectively delineate and enhance the presence of open water features in remotely sensed data (McFeeters, 1996). The index is designed to: (i) maximise the reflectance of water using green wavelengths (B3), (ii) minimize low reflectance of near infrared (B5) by water, and (iii) exploit the high reflectance of near infrared (B5) by vegetated and soil covered surfaces (Xu, 2006). Though oceans are masked, open water exists within the ice-free landscapes of Greenland as lakes and proglacial meltwater rivers. Many of the rivers have a strong suspended sediment content, making their visible spectral signal very similar to other features such as wet debris rich ice and saturated fine sediment facies. The NDWI improves the classifiers ability to differentiate between these features(Díaz-Delgado et al., 2006). The equation for NDWI is shown in equation (3).

The derived ratios are stacked as bands with the original 6 bands and subsequently mosaicked using a spectral weighting method derived from the near-infrared band (c.f. Hurni et al., 2017). Essentially the method strives to select only the best cell at each point for mosaicking. Six bands (Blue, Green, Red, NIR, SWIR1, SWIR2) and three derived ratios (NDVI, NDSI, NDWI) were selected from the mosaic and a Principal Component Analysis (PCA) conducted to reduce data dimensionality and band inter-correlation whilst maintaining >98% original variance. Despite the computational power of GEE, the complexity and computational cost of the pre-processing regime developed here required that image preparation be broken up into smaller regions, shown in Figure SI.2) around the periphery which are subsequently mosaicked together. Hundreds of Landsat scenes are processed meaning the highest quality final mosaicked image can be produced grid cell by grid cell, and free from shadow and cloud which is most unlikely to be achieved when using whole images / individual scenes. Coverage over Greenland is excellent for the contemporary dates, and for the late eighties is relatively good but with gaps in the south and west. Areas with no or bad data (e.g. cloud, shadow) present in both the eighties and contemporary imagery are masked from both images.

1. **Image Classification**


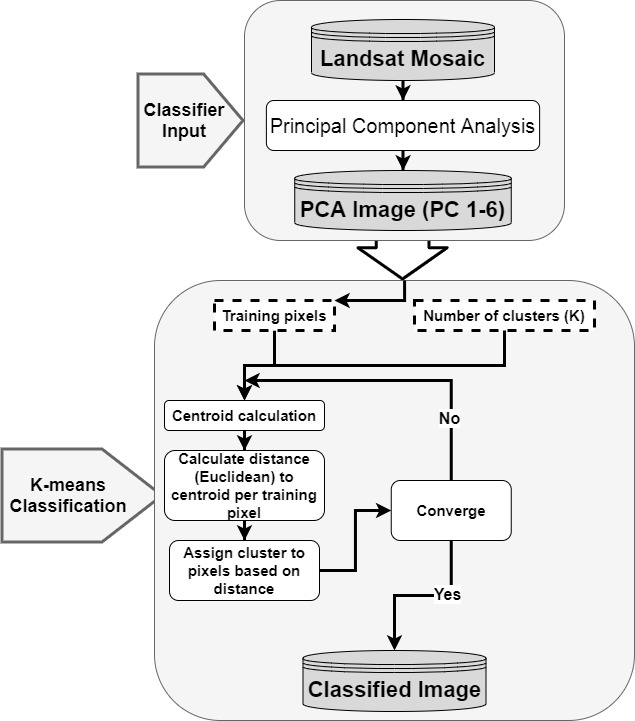


**Figure SI.4.** Unsupervised classification workflow for Landsat mosaics

| Table SI.1. Class Names and brief descriptions of class composition | |
| --- | --- |
| Class Name | Description |
| Bad data | Data which does not represent the earth surface i.e. cloud covered or hidden from analysis by shadow |
| Snow/Ice | Snow or ice, generally the Ice Sheet or ice caps and glaciers |
| Meltwater | Rivers which are more ice-proximal and have higher suspended sediment (SS) concentrations |
| Freshwater | Deep water bodies with lower suspended sediment (SS) such as lakes which appear darker, are lower energy or stagnant, or ice distal |
| Coarse sediment | Generally represents scree slopes, gravel beds and larger grained (>gravel) sediment facies |
| Fine-grained sediment | Lighter fine grained sediment beds which may predominantly be fluvial or aeolian. May also be weathered regolith in regions predominantly underlain by sandstone/sedimentary bedrock. |
| Bedrock | Bedrock exposures and heavily weathered bedrock. May also include some weathered regolith |
| Tundra vegetation | Vegetation which is comprised of sedges, mosses, grasses, dwarf shrubs and some sparse and scattered trees. |
| Dense/ wet vegetation | Dense established vegetation or those in wetlands with high TWI which may be composed of forests, meadows/farmland, or those specially adapted to live in bogs, fens and wetlands i.e. hydrophytes |

We produced multiple classifications using variable numbers of clusters, training pixels, training regions, and using both the original and principal component analysis images of the input mosaic, Comparison of those multiple classifications against other contemporary classifications from around Greenland (see: Jørgensen et al., 2015; Carrivick et al., 2017; Karami et al., 2018) and independent Sentinel-2A (10 m resolution) and PlanetScope (3 to 5 m resolution) images permitted us to determine that the best input and criteria were: K=70 clusters, 500,000 training pixels, and a PCA image of the input mosaic with the first 6 PC being selected. The PCA is used to reduce the dimensionality of the input data and to remove correlation between input bands. This is achieved via an eigenvector analysis of the correlation matrix of input bands, whereby axis of greatest variability between bands are identified and rotated to reduce obsolescence. The first 6 PC were then selected as the PCA indicated they contained the vast majority of significant information, over 98 % original bands variance. The best method determined for the contemporary classification was then also applied to the eighties mosaic for consistency.

Both unsupervised classifications were improved by conducting semi-supervised reclassifications to remove the greatest sources of error. The main miss-classifications were found between the Meltwater and Ice/snow classes and where shadows were misclassified as Freshwater. A Random Forest (RF) machine learning classifier was used in GEE to produce an ice mask, as wet ice and meltwater are largely spectrally indistinct (c.f. Breiman, 2001; Rodriguez-Galiano et al., 2012). Deep dark freshwater lakes are often misclassified as shadow, and vice vera. To reclassify misclassified freshwater as shadow, a simple slope threshold was used, whereby any cells classed as Freshwater with a slope angle over 12 degrees (extracted from ArcticDEM Mosaic) were re-classified to Bad Data (Shadow/Cloud). 12 degrees was chosen as the slope threshold by manual interrogation of misclassified shadow/lake regions. Both these methods were found to greatly improve the classification quality. The Random Forest (RF) classification workflow is outlined in Figure SI.5.


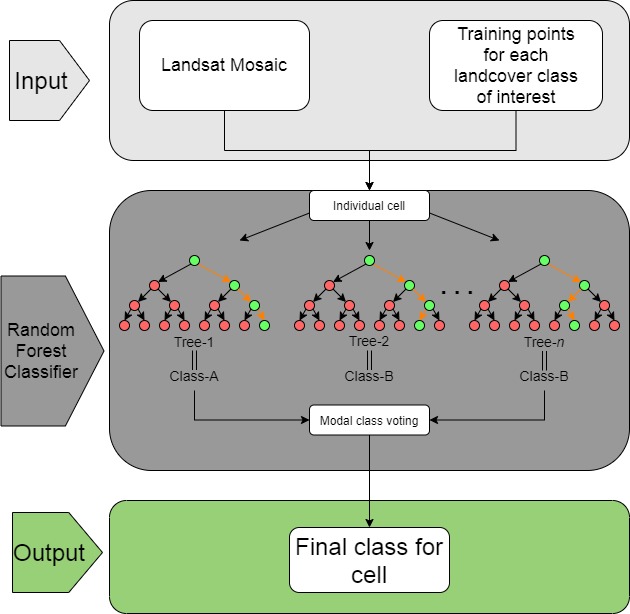


**Figure SI.5.** Random Forest RF classification method used for semi-supervised ice-mask production.

1. **Classification Accuracy Assessment**

Tables and equations referenced in, and further to, the information given in the methods section at the end of the main text.

| **Table SI.2.** Sample counts (*n_ij_*) correspondence matrix constructed from 3000 point stratified random sampling of contemporary semi-supervised classification. Classified map categories (*i*) are the rows and reference categories (*j*) are the columns. Grey shaded cells represent correct classification. *W_i_* is the area mapped of a class (*A_m,i_*) ÷ Total map area (*A_tot_*) | | | | | | | | | | | |
| --- | --- | --- | --- | --- | --- | --- | --- | --- | --- | --- | --- |
| **Class** | Snow/Ice | Meltwater/ Wet Ice | Freshwater | Coarse Sediment | Fine Sediment | Bedrock | Tundra Vegetation | Dense/ Wet Vegetation | Total (*n_i·_*) | Total area (km^2^) | *W_i_* |
| Snow/Ice | 584 | 23 | 9 | 0 | 2 | 0 | 0 | 0 | 618 | 254,527.32 | 0.206 |
| Meltwater/ Wet Ice | 16 | 127 | 12 | 2 | 3 | 0 | 0 | 1 | 161 | 66,214.41 | 0.054 |
| Freshwater | 3 | 11 | 46 | 2 | 0 | 0 | 0 | 2 | 64 | 26,422.66 | 0.021 |
| Coarse Sediment | 5 | 13 | 4 | 469 | 35 | 51 | 1 | 2 | 580 | 239,257.86 | 0.193 |
| Fine Sediment | 2 | 9 | 0 | 24 | 147 | 36 | 2 | 0 | 220 | 86,847.04 | 0.070 |
| Bedrock | 1 | 5 | 12 | 89 | 64 | 768 | 16 | 13 | 968 | 403,232.11 | 0.326 |
| Tundra Vegetation | 0 | 0 | 3 | 7 | 4 | 3 | 283 | 28 | 328 | 135,202.45 | 0.109 |
| Dense/Wet Vegetation | 0 | 0 | 0 | 2 | 1 | 2 | 9 | 47 | 61 | 24,989.45 | 0.020 |
| Total (*n_j·_*) | 611 | 188 | 86 | 595 | 256 | 860 | 311 | 93 | 3000 | 1,236,693.29 | 0.206 |

| **Table SI.3.** Correspondence matrix (Table SI.2.) where cells are expressed as the estimated proportion of area (*p̂_ii_* Classified map categories (*i*) are the rows and reference categories (*j*) are the columns. | | | | | | | | | | | |
| --- | --- | --- | --- | --- | --- | --- | --- | --- | --- | --- | --- |
| **Class** | Snow/  Ice | Meltwater/ Wet Ice | Freshwater | Coarse Sediment | Fine Sediment | Bedrock | Tundra Vegetation | Dense/ Wet Vegetation | Total (*p̂·_i_*) | *Û_i_* | *p̂_j_* |
| Snow/ Ice | 0.1945 | 0.0077 | 0.0030 | 0.0000 | 0.0007 | 0.0000 | 0.0000 | 0.0000 | 0.2058 | 0.9450 | 0.9559 |
| Meltwater/ Wet Ice | 0.0053 | 0.0422 | 0.0040 | 0.0007 | 0.0010 | 0.0000 | 0.0000 | 0.0003 | 0.0535 | 0.7888 | 0.6762 |
| Freshwater | 0.0010 | 0.0037 | 0.0154 | 0.0007 | 0.0000 | 0.0000 | 0.0000 | 0.0007 | 0.0214 | 0.7188 | 0.5347 |
| Coarse Sediment | 0.0017 | 0.0043 | 0.0013 | 0.1564 | 0.0117 | 0.0170 | 0.0003 | 0.0007 | 0.1935 | 0.8086 | 0.7885 |
| Fine Sediment | 0.0006 | 0.0029 | 0.0000 | 0.0077 | 0.0469 | 0.0115 | 0.0006 | 0.0000 | 0.0702 | 0.6682 | 0.5621 |
| Bedrock | 0.0003 | 0.0017 | 0.0040 | 0.0300 | 0.0216 | 0.2587 | 0.0054 | 0.0044 | 0.3261 | 0.7934 | 0.8956 |
| Tundra Vegetation | 0.0000 | 0.0000 | 0.0010 | 0.0023 | 0.0013 | 0.0010 | 0.0943 | 0.0093 | 0.1093 | 0.8628 | 0.9099 |
| Dense/Wet Vegetation | 0.0000 | 0.0000 | 0.0000 | 0.0007 | 0.0003 | 0.0007 | 0.0030 | 0.0156 | 0.0202 | 0.7705 | 0.5031 |
| Total (*p̂·_j_*) | 0.2035 | 0.0625 | 0.0287 | 0.1984 | 0.0835 | 0.2889 | 0.1037 | 0.0309 | 1.0000 | 6.3560 | 5.8259 |

Table SI.2. is represented in Table SI.3. as estimators of proportional area in each cell *i,j* of the matrix, with each value being calculated using equations 4 through 7:

|  | $\hat{p}ij=W_{i}\frac{p_{ii}}{p_{i}}$ | (4) |
| --- | --- | --- |
|  | $\hat{U_{i}}=User error=\frac{p_{ii}}{p_{i}}$ | (5) |
|  | $\hat{p}j=Producer error=\frac{p_{jj}}{p_{j}}$ | (6) |
|  | $\hat{O}=Overall accuracy= \sum_{j=1}^{8} p_{ij}=\boldsymbol{0.824}$ | (7) |

Total proportional area accounting for stratified errors can be calculated for each class as:

|  | $\sum_{i}^{8} W_{i}\frac{n_{ij}}{n_{i}}$ | (8) |
| --- | --- | --- |

The stratified error-adjusted class estimator of area *Â_j_* is calculated as:

|  | $\hat{A_{j}}=A_{tot}\hat{p}_{j}$ | (9) |
| --- | --- | --- |

Error margins (confidence intervals) for *Â_j_* are calculated based on sensitivity to the users and producers errors shown in Table SI.3 as a standard error, calculated as:

|  | $S(\hat{p_{j}})=\sqrt{\sum_{i=1}^{8} W_{i}^{2}}\frac{\frac{n_{ij}}{n_{i}}\left( 1-\frac{n_{ij}}{n_{i}} \right)}{n_{i}-1}$ | (9) |
| --- | --- | --- |

The confidence intervals expressed at a 95% error margin (z-score = 1.96) are calculated as:

|  | $\hat{A}_{j}=\pm1.96\times S(p̂\cdot j)$ | (9) |
| --- | --- | --- |

Stratified area estimates for each class with 95% confidence intervals as error margins are displayed in terms of percentage of total are in Table SI.4.

| **Table SI. 4.** Landcover class stratified area estimates and 95% error margins | | | | |
| --- | --- | --- | --- | --- |
| **Class** | ***Stratified ‘error adjusted’ areas***  ***Â·_j_ (km^2^)*** | ***95% confidence interval***  ***1.96*×*S(p̂·_j_)(km^2^)*** | ***Â·_j_ %Total area*** | ***95% confidence interval***  ***%*** |
| Snow/Ice | 251,611.72 | ± 6,115.46 | 20.35 % | ± 2.43% |
| Meltwater/ Wet Ice | 77,243.66 | ± 7,408.17 | 6.25 % | ± 9.59% |
| Freshwater | 35,518.64 | ± 5,837.44 | 2.87 % | ± 16.43% |
| Coarse Sediment | 245,370.10 | ± 11,571.44 | 19.84 % | ± 4.72% |
| Fine Sediment | 103,243.55 | ± 9,859.61 | 8.35 % | ± 9.55% |
| Bedrock | 357,225.15 | ± 12,556.56 | 28.89 % | ± 3.52% |
| Tundra Vegetation | 128,207.33 | ± 6,541.80 | 10.37 % | ± 5.10% |
| Dense/Wet Vegetation | 38,273.15 | ± 5,969.91 | 3.09 % | ± 15.60% |

**Change Area and Confidence Intervals**

The change in total coverage was simply calculated as the contemporary error-adjusted class area (contemp Â·_j_) minus the 1980s error-adjusted areas (eighties Â·_j_). Confidence intervals (CI) for changes in area are calculated as the square root of the sum-of-square error for each period, divided by the average area between the two periods. Table SI. 5 shows the error adjusted areas for each period, the change area (in km^2^ and percent), average area between each period, and the change confidence intervals (in km^2^ and percent).

| Table SI. 5. Landcover class change statistics as reported in main manuscript | | | | | | |
| --- | --- | --- | --- | --- | --- | --- |
| Class | **1980s Error-adjusted Area (km^2^)** | **Contemporary Error-adjusted Area (km^2^)** | **Change Area (km^2^)** | **Percent Change (%)** | **Change CI Area (km^2^)** | **Change CI Percent (%)** |
| Ice | 251,611.72 | 280,318.82 | -28,707.10 | -10.24 | 9,766.86 | 3.67 |
| Meltwater | 77,243.66 | 67,212.65 | 10,031.00 | 14.92 | 10,516.63 | 14.56 |
| Freshwater | 35,518.64 | 39,854.54 | -4,335.90 | -10.88 | 8,420.79 | 22.34 |
| Coarse Sediment | 245,370.10 | 246,553.43 | -1,183.34 | -0.48 | 15,454.49 | 6.28 |
| Fine Sediment | 103,243.55 | 98,969.49 | 4,274.06 | 4.32 | 13,238.49 | 13.09 |
| Bedrock | 357,225.15 | 424,778.44 | -67,553.29 | -15.90 | 16,681.75 | 4.27 |
| Tundra Vegetation | 128,207.33 | 71,027.30 | 57,180.03 | 80.50 | 8,557.83 | 8.59 |
| Wetland Vegetation | 38,273.15 | 7,978.61 | 30,294.54 | 379.70 | 6,805.40 | 29.43 |
| Vegetation Combined | 166,480.48 | 79,005.91 | 87,474.57 | 110.72 | 15,287.54 | 12.45 |

**Field Validation**

Figure SI.6. shows the detail of sites where field validation was conducted, namely panels of the aerodem images, RGB, NGB and the contemporary classification with validation points overlaid. Validation conducted in panels A – D (Figure SI.6) was collected during fieldwork in August 2022, and validation around Zackenberg in panel E was collected during August 2017. Figure SI.7. shows a series of photos collected during fieldwork with reference to landcover as classified and observed, location (Lat, Long in Decimal Degrees), and a brief description of the land cover.


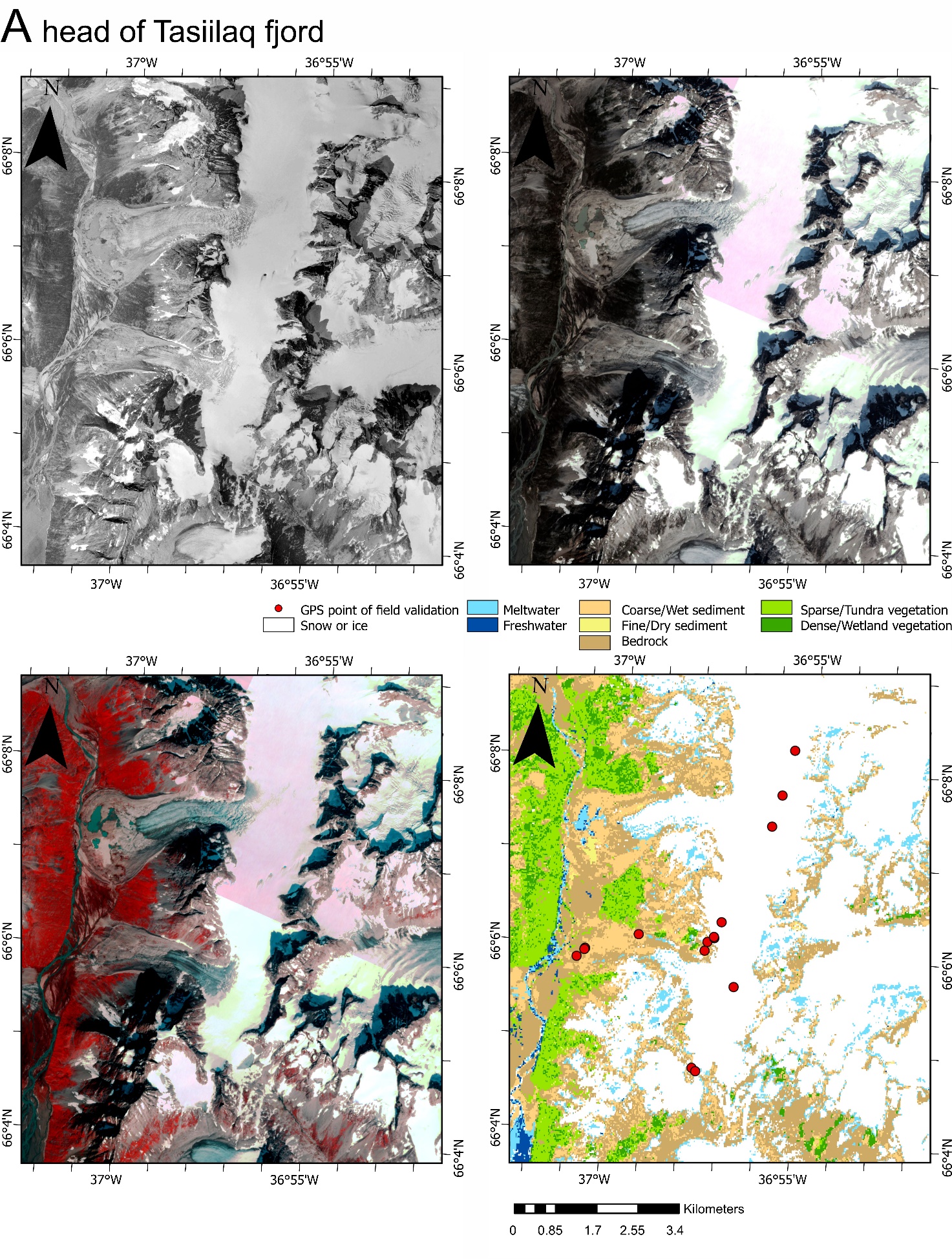


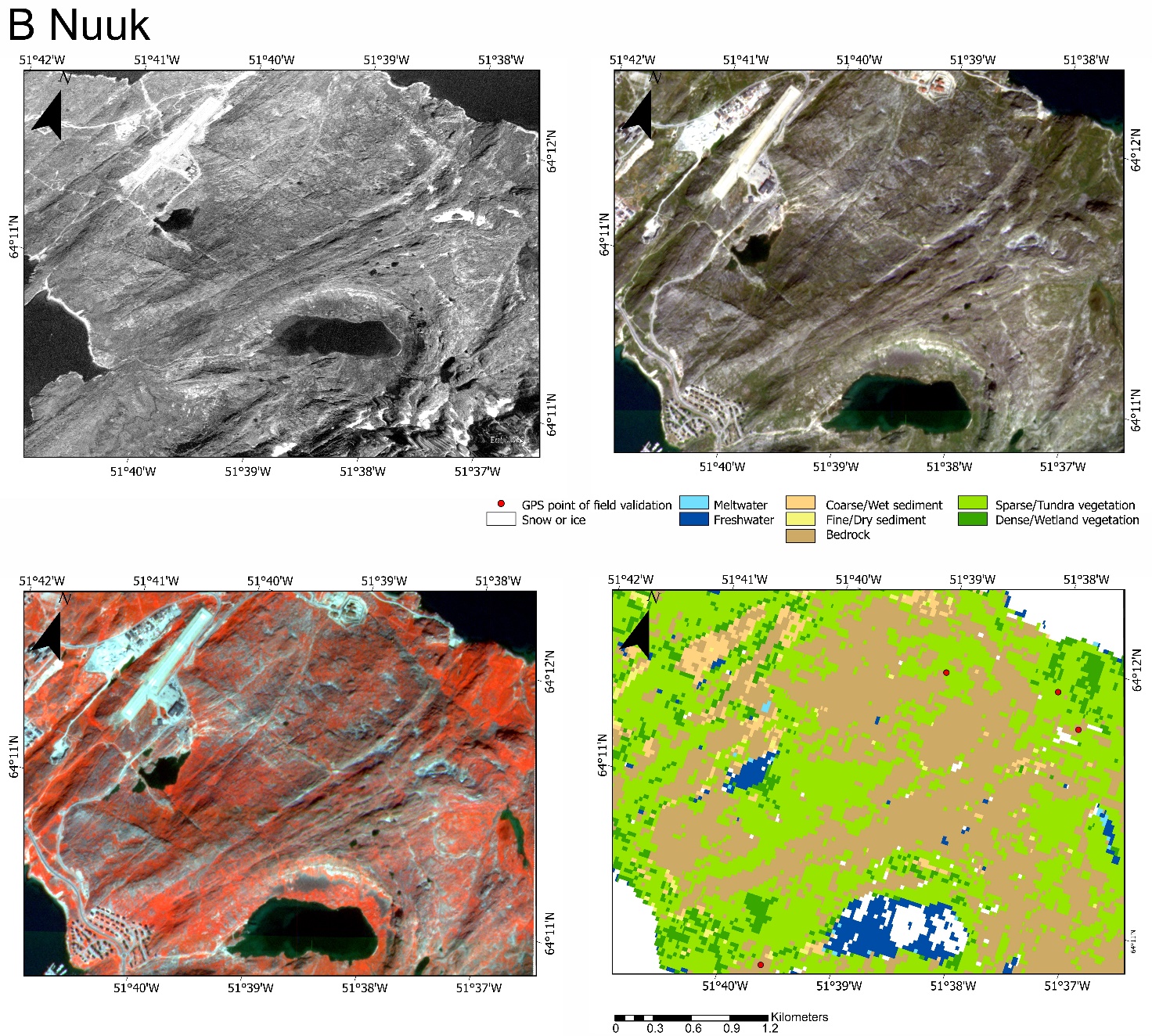


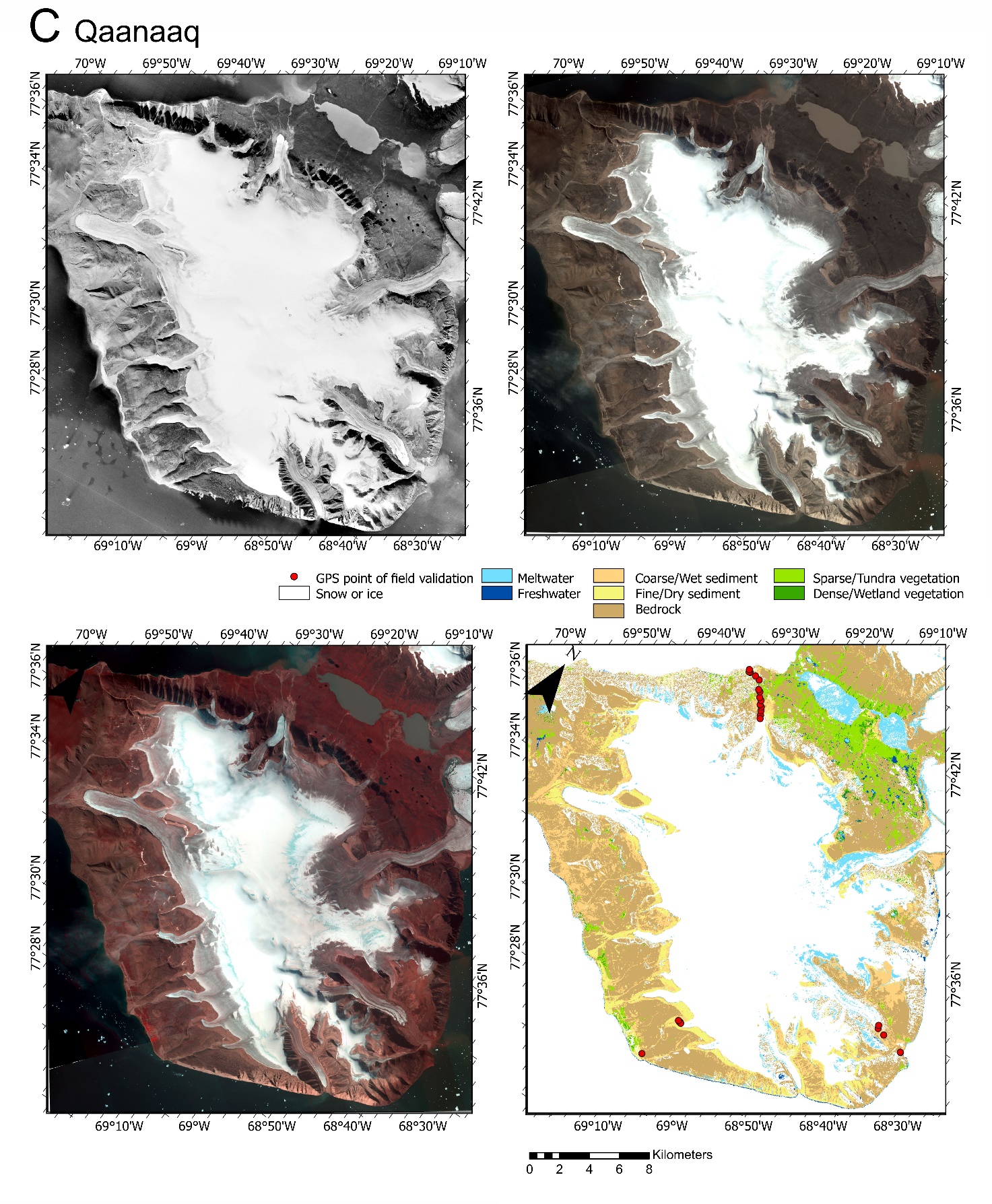


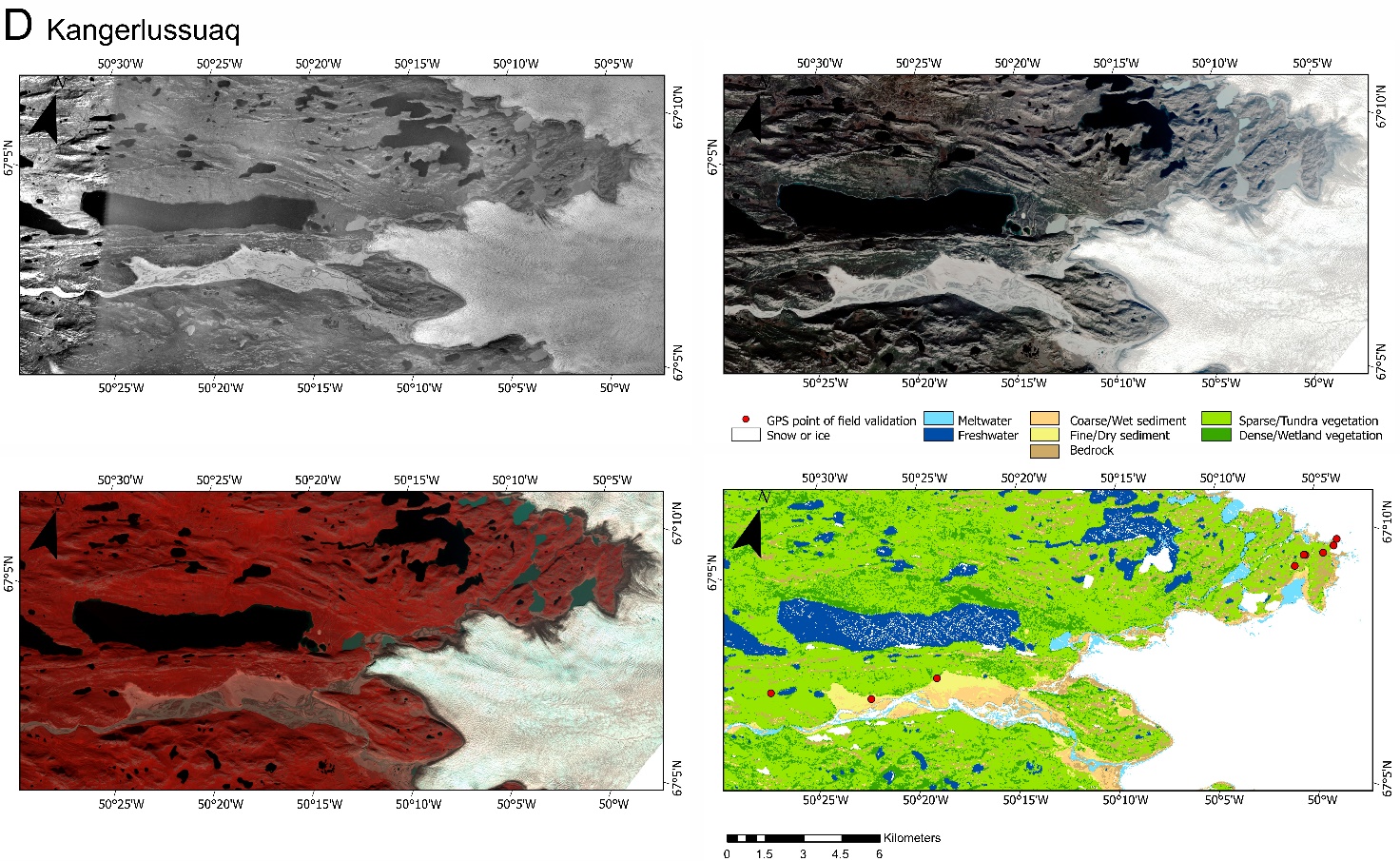


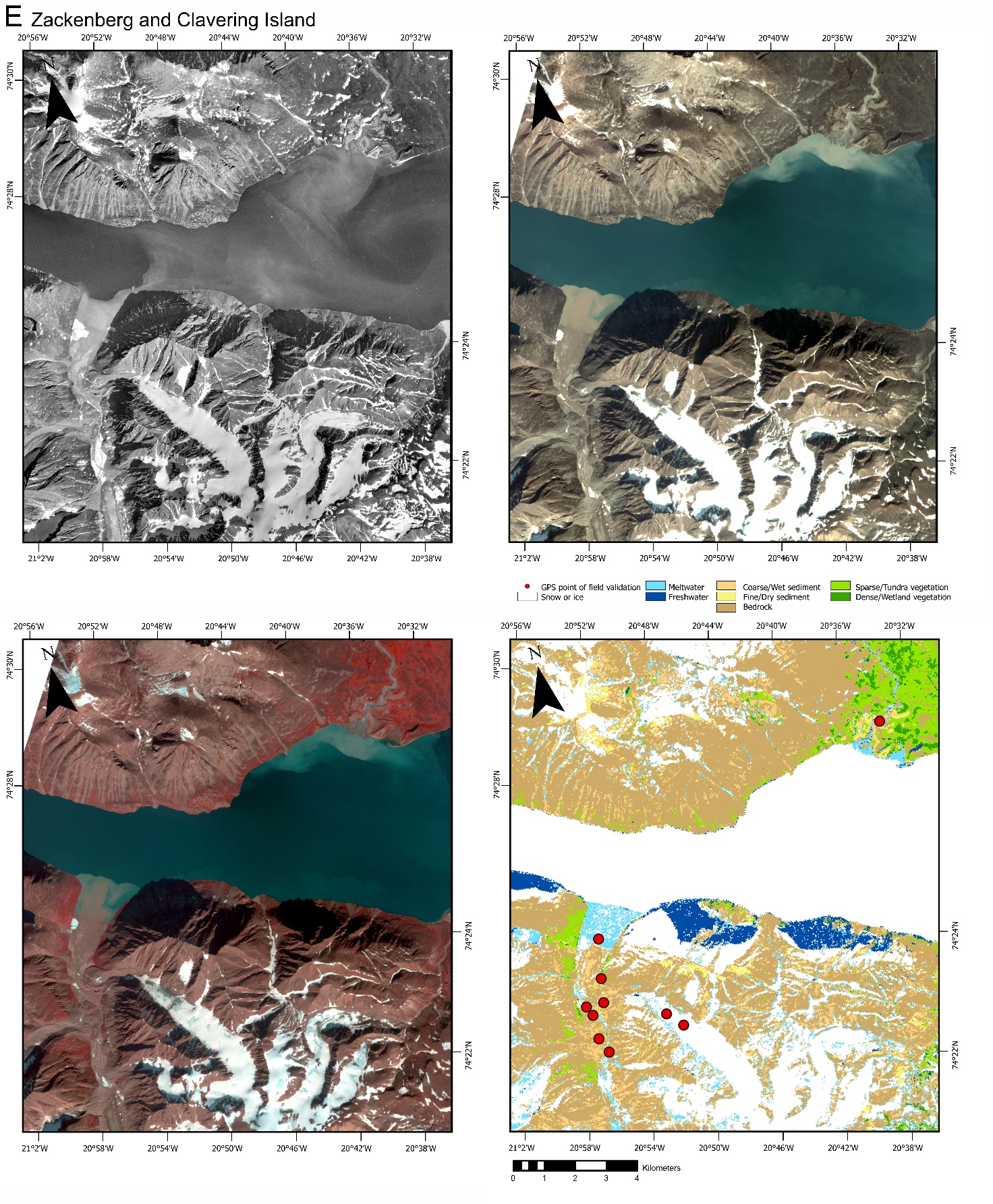


Figure SI.6. Examples of images used towards validation. We highlight 5 locations where we also have field observations (red points) as depicted in Fig. SI.7. Each location is indicated in Fig. 1. The four panels for each location comprise an aerial photograph (1980s), a RGB Planet image (2019), a NGB Planet image (2019) and our resultant land cover classification.

**Panel A: Tasiilaq Mountain Hut**

**Lat/Long: N66.105, W -36.953**

**Classified: Snow/Ice**

**Validated: Ice**


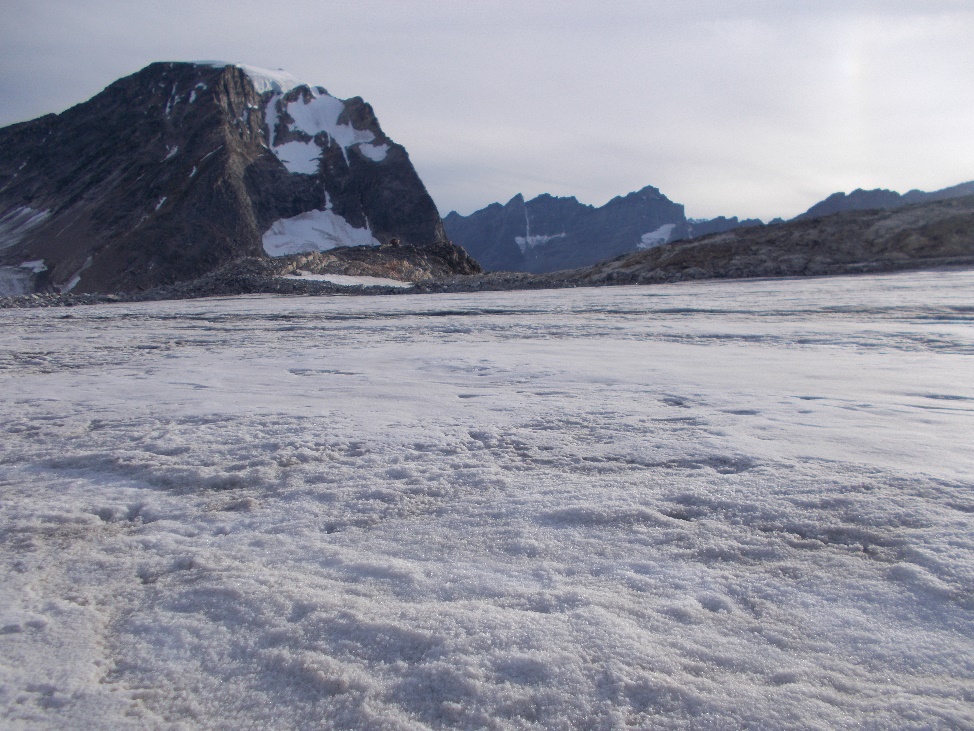


**Panel B: Nuuk**

**Lat/Long: N64.196, W -51.625**

**Classified: Tundra Vegetation**

**Validated: Tundra Vegetation**


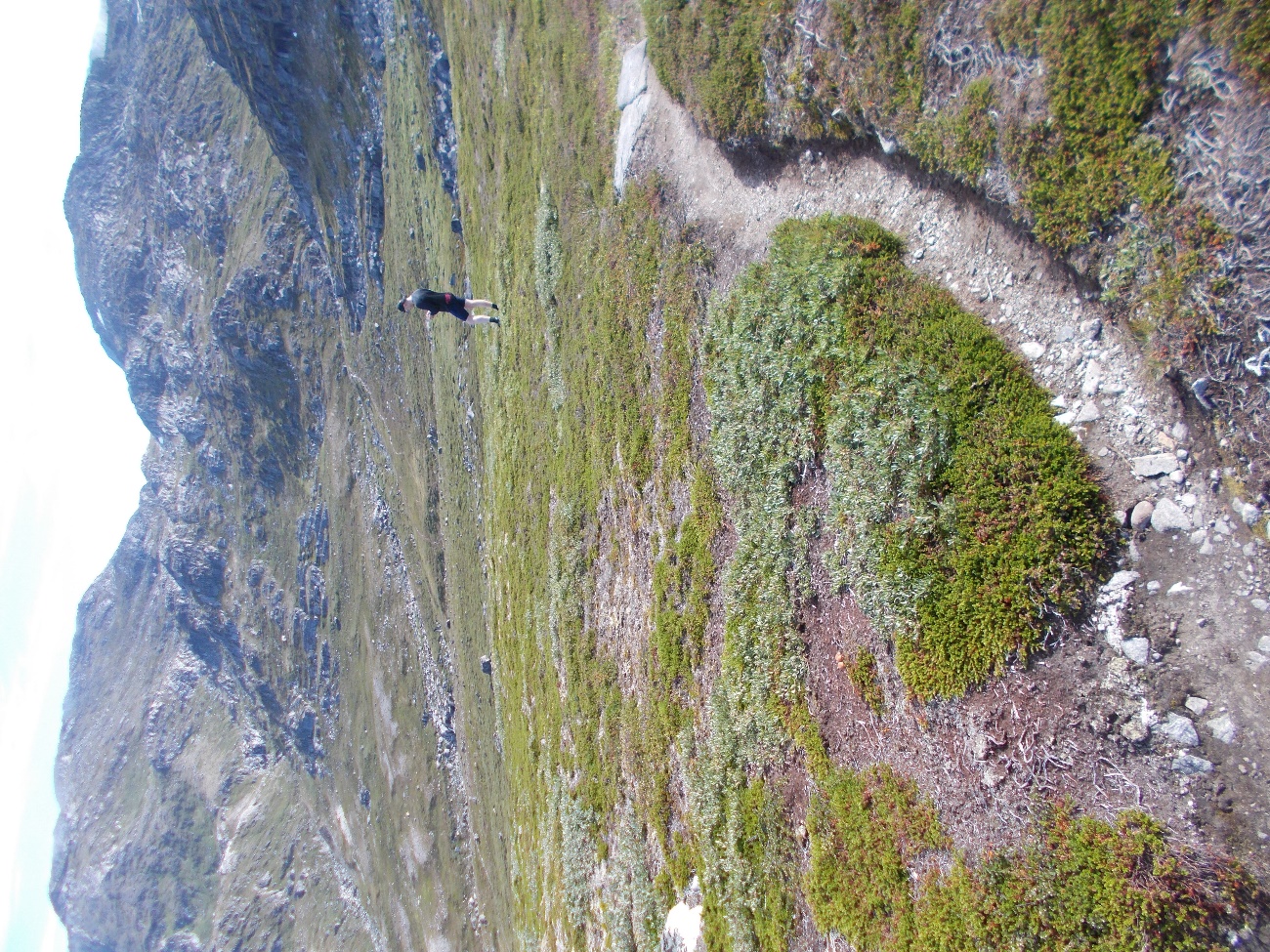


**Panel B: Nuuk**

**Lat/Long: N64.197, W -51.648**

**Classified: Tundra Vegetation**

**Validated: Tundra Vegetation**


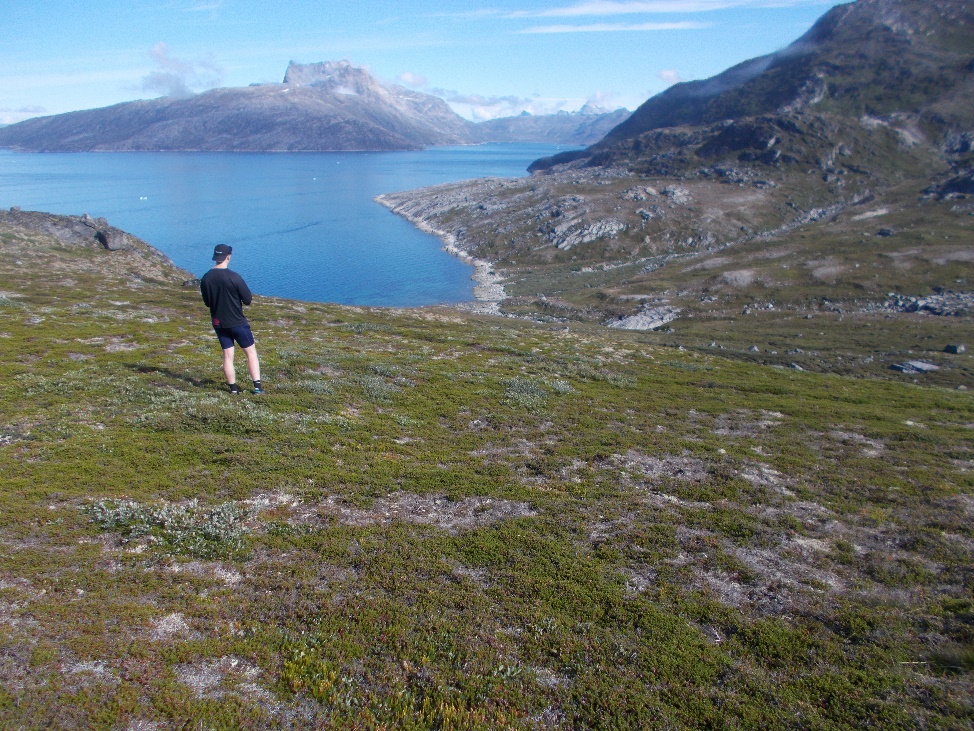


**Panel B: Nuuk**

**N64.1737, W -51.654**

**Classified: Bedrock**

**Validated: Weathered Bedrock**


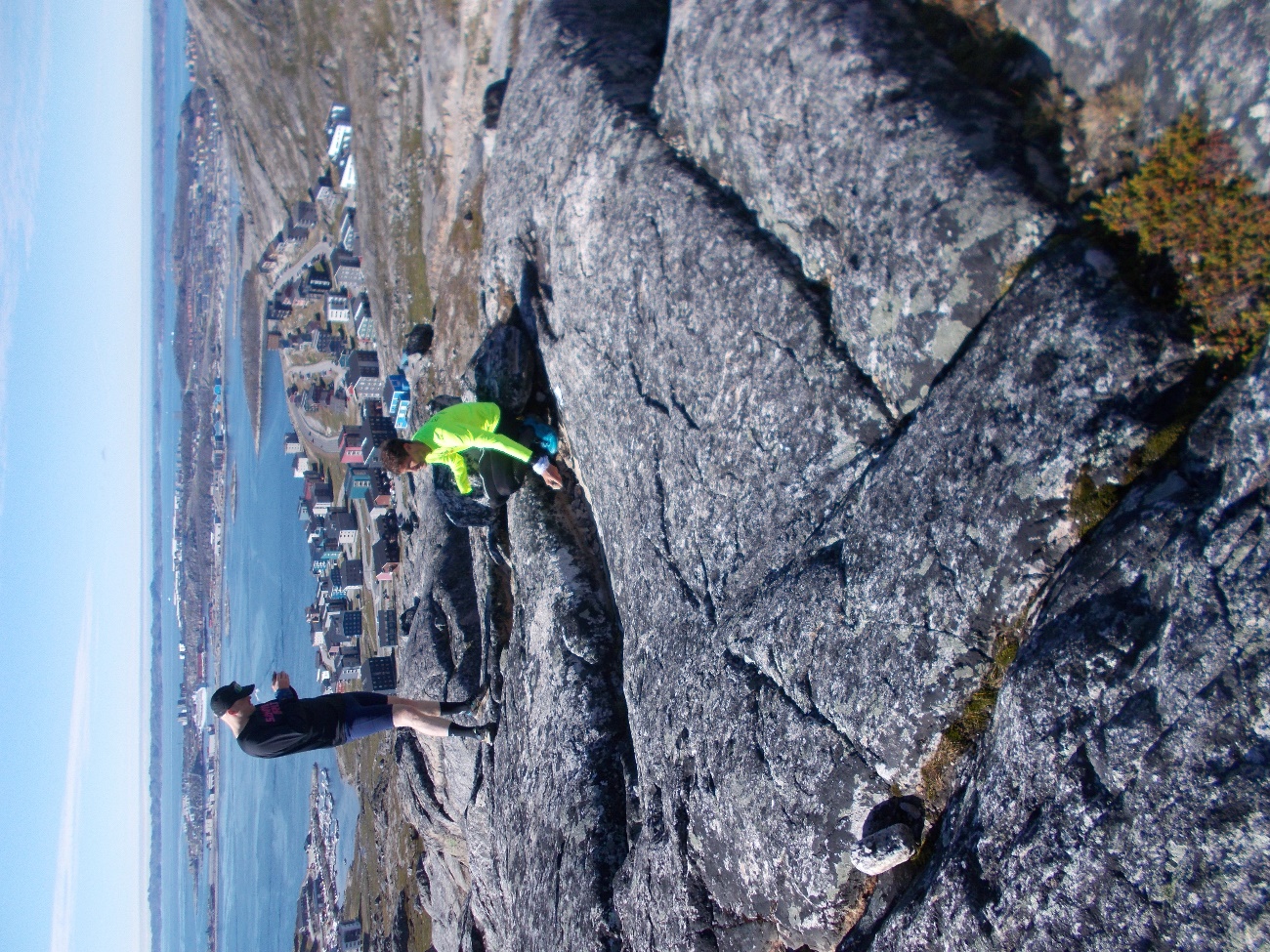


**Panel B: Nuuk**

**Lat/Long: N64.1739, W -51.661**

**Classified: Bedrock**

**Validated: Abraded Bedrock**


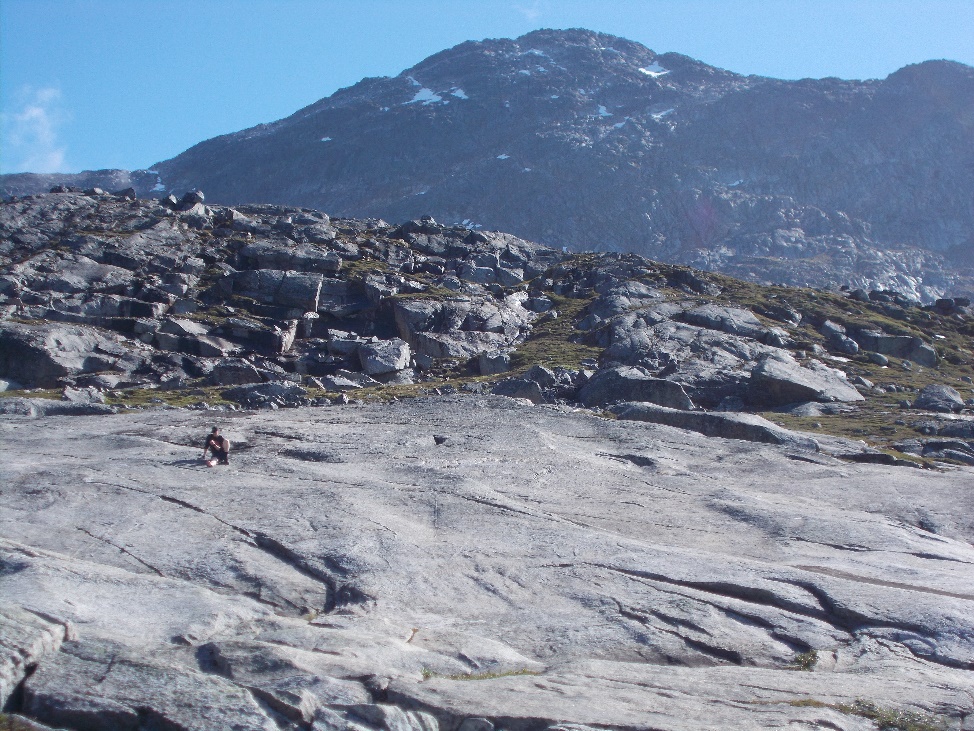

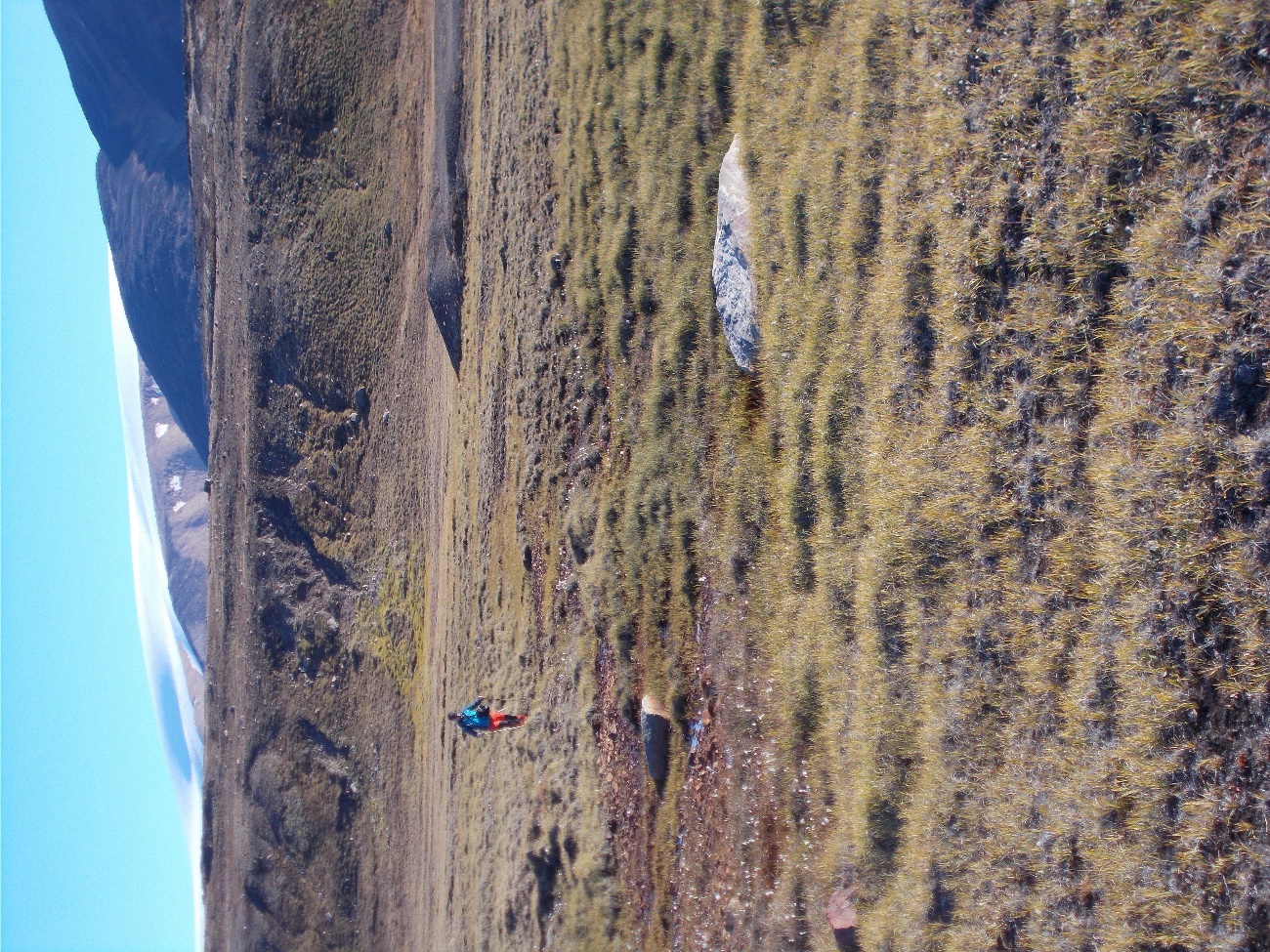


**Panel C: Qaanaaq**

**Lat/Long: N77.681, W -69.521**

**Classified: Tundra Vegetation**

**Validated: Tundra Vegetation**

**Panel C: Qaanaaq**

**Lat/Long: N77.668, W -69.457**

**Classified: Bedrock**

**Validated: Large boulders, coarse sediment**


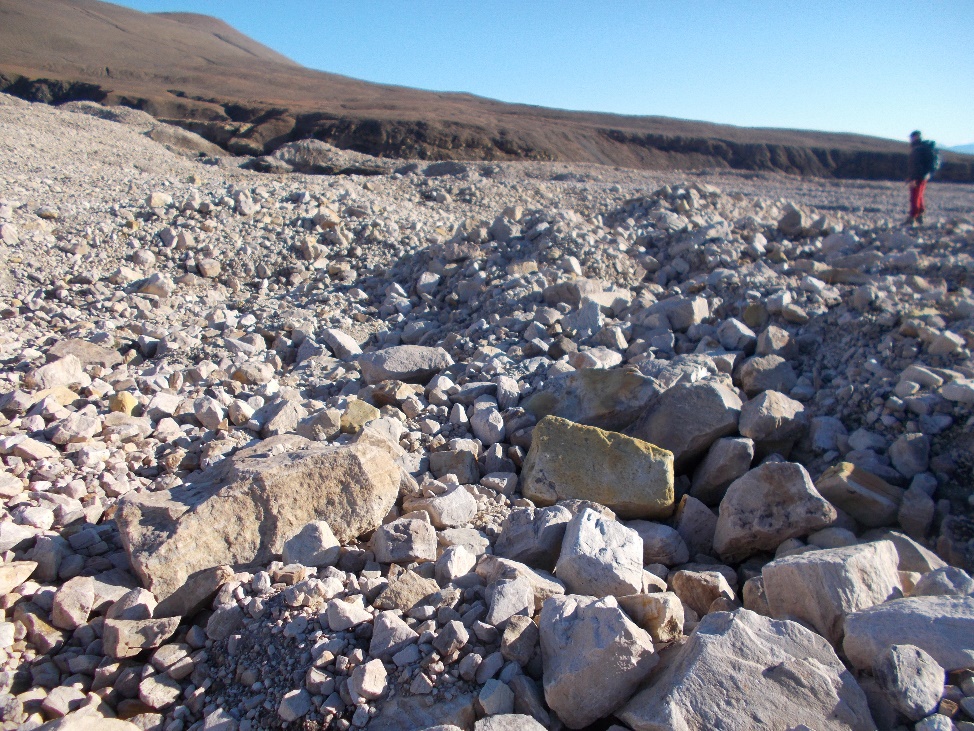

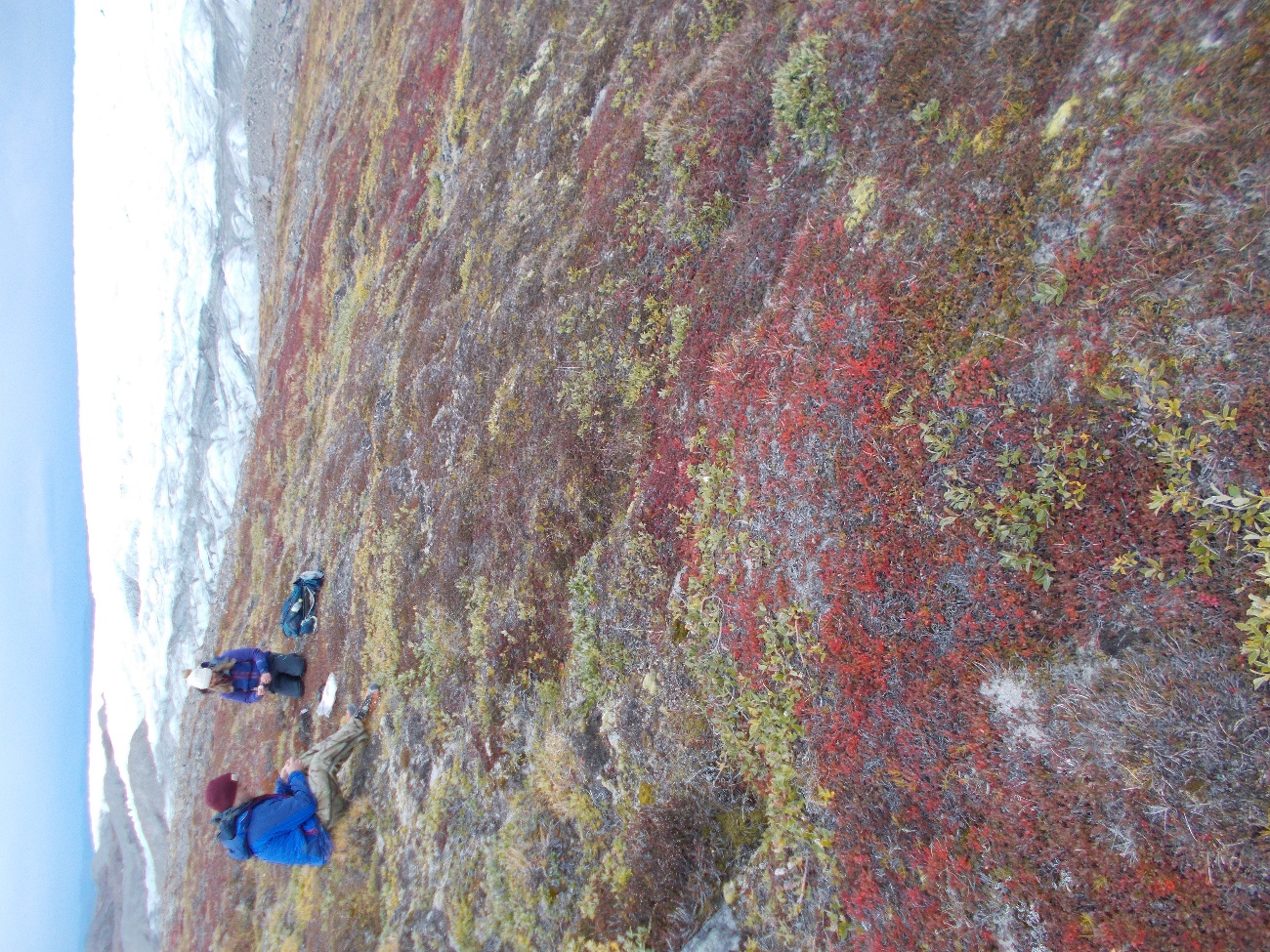


**Panel D: Kangerlussuaq, Russel Glacier**

**Lat/Long: N 67.151, W -50.074**

**Classified: Tundra Vegetation**

**Validated: Tundra Vegetation**


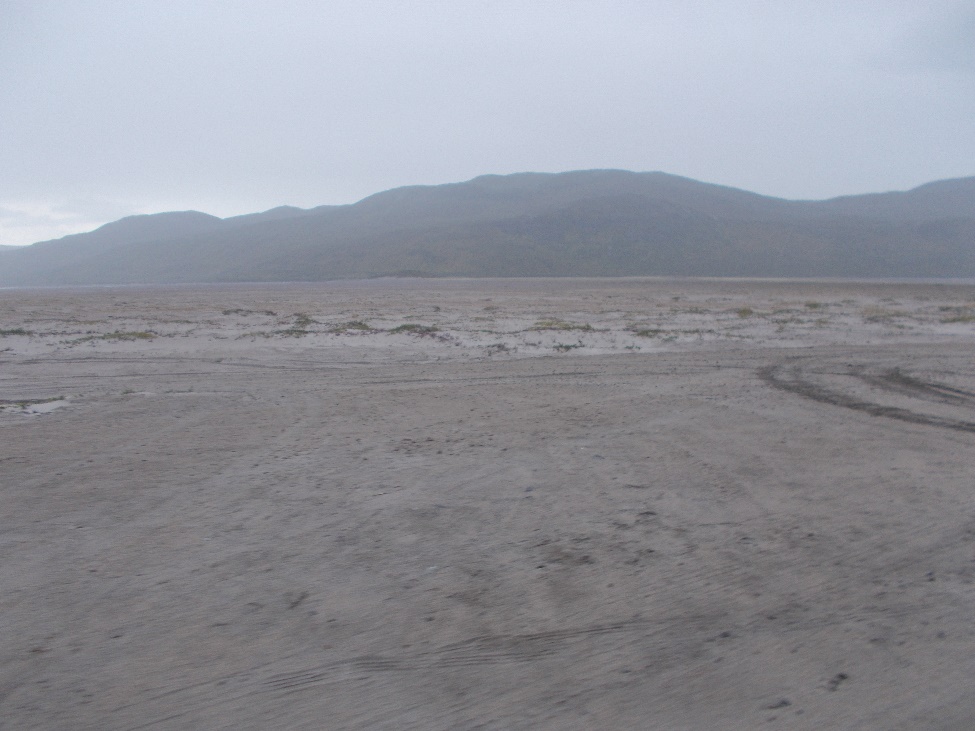


**Panel D: Kangerlussuaq, Sandflugtdalen**

**Lat/Long: N 67.073, W -50.349**

**Classified: Fine Sediment**

**Validated: Fine Sediment, Sand**


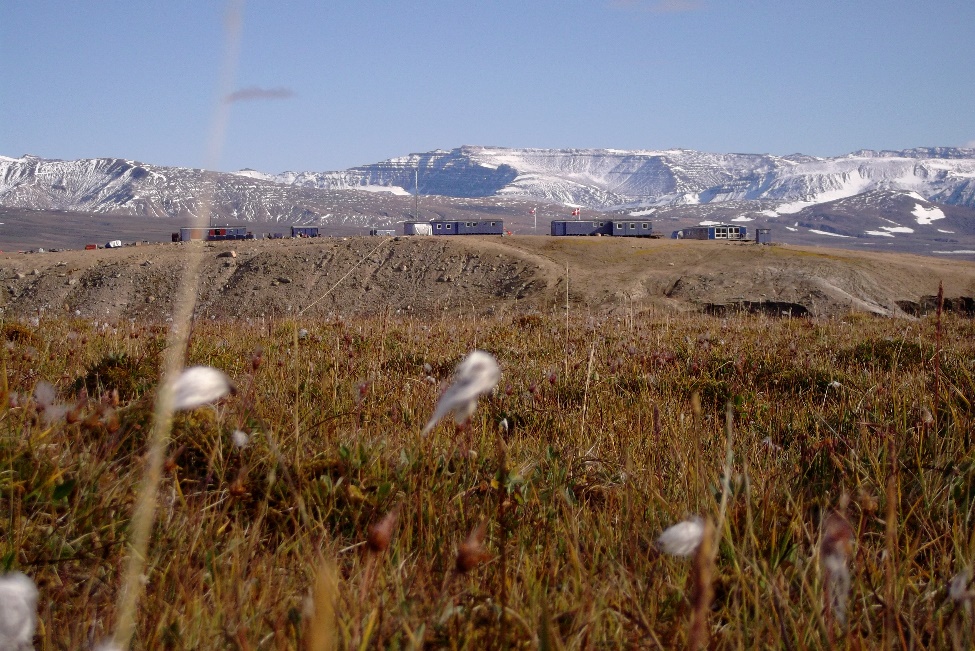


**Panel E: Zackenberg**

**Lat/Long: N 74.468, W -20.589**

**Classified: Wetland Vegetation**

**Validated: Tundra vegetation on regularly flooded ground**


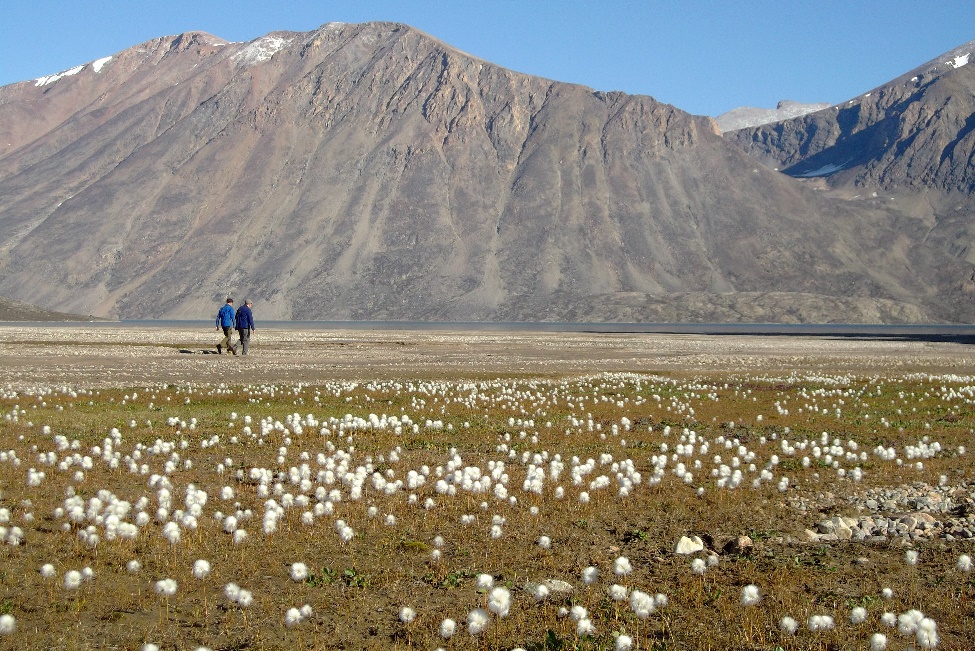


**Panel E: Clavering Island**

**Lat/Long: N 74.414, W -20.937**

**Classified: Tundra Vegetation**

**Validated: Tundra vegetation**

Figure SI.7. Photographs taken during field validation. Panel letters refer to panels shown in Figure SI.6.

**Panel E: Clavering Island**

**Lat/Long: N 74.397, W -20.953**

**Classified: Coarse sediment**

**Validated: Coarse sediment, moraine/till**


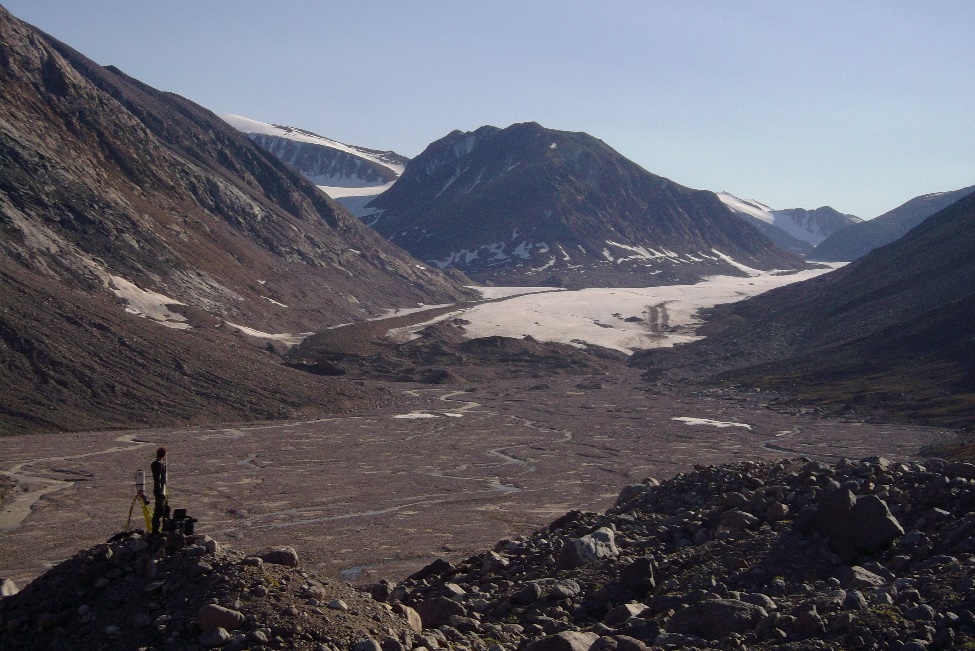


Overall, 75 of the 89 field validation points were correctly classified based on ground validation. This equates to 84% accuracy, and the confusion matrix indicated an overall Kappa coefficient of 0.81. Misclassifications were largely proglacial moraine being classified as bedrock, and dry tundra vegetation being classified as wetland.

**Classification change accuracy assessment**

Here we present the tables relating to our classification change accuracy assessment as outlined in methods of the main text. Table SI.6. show the legend describing the 64 change class designations, and table SI.7. shows the mode of class aggregation.

| 64 class image values | From Class |
| --- | --- |
| 0 - 7 | Snow/Ice |
| 8 - 15 | Meltwater |
| 16 - 23 | Freshwater |
| 24 - 31 | Coarse/dark Sedment |
| 32 - 39 | Fine-grained sediment |
| 40 - 47 | Bedrock |
| 48 - 55 | Dry tundra |
| 56 - 63 | Dense/wet Vegetation |
| Table SI 6. Legend for 64 class (values from 0 – 63). From class represent the class at that location in the 1980s and the values within represent changes from that class to itself (static) and the other 7 classes. E.g. Change class value 0 = change from *Snow/Ice* to *Snow/Ice* (static), change class value 1 = change from *Snow/Ice* to *Meltwater*, change class value 8 = change from *Meltwater* to. *Snow/Ice*, change class value 62 = change from *Dry tundra* to *Dense/wet vegetation,* Etc. | |

| Aggregation class description | Aggregation class value | 64 class value |
| --- | --- | --- |
| Snow/Ice to Snow/Ice | 1 | 1 |
| Snow/Ice to Water | 2 | 2,3 |
| Snow/Ice to Barren* Ground | 3 | 4,5,6 |
| Snow/Ice to Vegetation | 4 | 7,8 |
| Water to Snow/Ice | 5 | 9,17 |
| Water to Water | 6 | 10,11,18,19 |
| Water to Barren* Ground | 7 | 12,13,14,20,21,22 |
| Water to Vegetation | 8 | 15,16,23,24 |
| Barren* Ground to Snow/Ice | 9 | 25,33,41 |
| Barren* Ground to Water | 10 | 26,27,34,35,42,43 |
| Barren* Ground to Barren* Ground | 11 | 28,29,30,36,37,38,44,45,46 |
| Barren* Ground to Vegetation | 12 | 31,32,39,40,47,48 |
| Vegetation to Snow/Ice | 13 | 49,57 |
| Vegetation to Water | 14 | 50,51,58,59 |
| Vegetation to Barren* Ground | 15 | 52,53,54,60,61,62 |
| Vegetation to Vegetation | 16 | 55,56,63,64 |
| Table SI 7. Change class aggregation schema. Aggregation rules: Snow/Ice has no aggregation, Meltwater and Deep Freshwater became Water, unconsolidated sediment classes and bedrock became Barren Ground, Dry Tundra vegetation and wet/dense vegetation became Vegetation. * Barren is defined as unvegetated bare earth, not perennially covered by snow, ice, or water | | |

Recent developments in change class accuracy assessment have proposed and explored local versions of accuracy measures (overall accuracy, user’s Type I or commission error accuracy, and producer’s Type II or omission error accuracy), in order to understand the spatial distribution of different levels of different types of accuracy in the study area. A series of paper have developed geographically weighted approaches to error reporting as outlined in Foody (2005) and then developed in detail in A. Comber et al. (2012), A. J. Comber (2013) and A. Comber et al. (2017). In brief these use a moving window or a kernel and compute a series of local correspondence matrices from which local accuracy measures are generated. Here the approach of A. J. Comber (2013) is adopted, linking the derivation of overall, user’s and producer’s accuracy to standard generalized, binomial regression, which can then be easily related to the spatial case via geographically weighted binomial regression.

A cross tabulation of predicted against observed class is shown in Table SI 8. From this overall and class specific accuracies can be derived (Congalton 1991) as shown in Table SI 9.

| Agg. Class | 1 | 2 | 3 | 4 | 5 | 6 | 7 | 8 | 9 | 10 | 11 | 12 | 13 | 14 | 15 | 16 |
| --- | --- | --- | --- | --- | --- | --- | --- | --- | --- | --- | --- | --- | --- | --- | --- | --- |
| 1  Snow/Ice to Snow/Ice | 172 | 0 | 1 | 0 | 0 | 0 | 0 | 0 | 0 | 0 | 0 | 0 | 0 | 0 | 0 | 0 |
| 2  Snow/Ice to Water | 35 | 124 | 8 | 0 | 0 | 4 | 0 | 0 | 0 | 0 | 1 | 0 | 0 | 0 | 0 | 0 |
| 3  Snow/Ice to Barren* Ground | 0 | 2 | 285 | 0 | 0 | 0 | 0 | 0 | 0 | 0 | 43 | 0 | 0 | 0 | 0 | 0 |
| 4  Snow/Ice to Vegetation | 0 | 0 | 0 | 13 | 0 | 0 | 0 | 0 | 0 | 0 | 1 | 5 | 0 | 0 | 0 | 4 |
| 5  Water to Snow/Ice | 7 | 0 | 0 | 0 | 11 | 9 | 0 | 0 | 0 | 0 | 0 | 0 | 0 | 0 | 0 | 0 |
| 6  Water to Water | 35 | 4 | 4 | 0 | 0 | 260 | 0 | 0 | 0 | 0 | 19 | 0 | 0 | 0 | 0 | 0 |
| 7  Water to Barren* Ground | 1 | 0 | 13 | 0 | 0 | 7 | 114 | 0 | 0 | 0 | 57 | 0 | 0 | 0 | 0 | 0 |
| 8  Water to Vegetation | 0 | 0 | 0 | 1 | 0 | 0 | 0 | 7 | 0 | 0 | 0 | 1 | 0 | 0 | 0 | 1 |
| 9  Barren* Ground to Snow/Ice | 8 | 1 | 0 | 0 | 0 | 0 | 0 | 0 | 55 | 5 | 17 | 2 | 0 | 0 | 0 | 0 |
| 10  Barren* Ground to Water | 0 | 0 | 0 | 0 | 0 | 2 | 0 | 0 | 15 | 134 | 43 | 5 | 0 | 0 | 0 | 0 |
| 11  Barren* Ground to Barren* Ground | 0 | 0 | 0 | 0 | 0 | 0 | 0 | 0 | 0 | 1 | 527 | 21 | 0 | 0 | 0 | 0 |
| 12  Barren* Ground to Vegetation | 0 | 0 | 0 | 0 | 0 | 0 | 0 | 0 | 0 | 0 | 10 | 388 | 0 | 0 | 0 | 27 |
| 13  Vegetation to Snow/Ice | 1 | 0 | 0 | 0 | 0 | 0 | 0 | 0 | 0 | 0 | 0 | 0 | 2 | 0 | 0 | 0 |
| 14  Vegetation to Water | 0 | 0 | 0 | 0 | 0 | 5 | 0 | 0 | 0 | 0 | 2 | 0 | 0 | 16 | 0 | 7 |
| 16  Vegetation to Barren* Ground | 0 | 0 | 0 | 0 | 0 | 0 | 0 | 0 | 0 | 0 | 7 | 0 | 0 | 0 | 31 | 19 |
| 17  Vegetation to Vegetation | 0 | 0 | 0 | 0 | 0 | 0 | 0 | 0 | 0 | 1 | 0 | 9 | 0 | 0 | 0 | 189 |
| Table SI 8. The cross tabulation of predicted (rows) and observed (columns). | | | | | | | | | | | | | | | | |

| Agg. class | Users | Producers | Overall | Kappa |
| --- | --- | --- | --- | --- |
| 1  Snow/Ice to Snow/Ice | 0.994 | 0.664 | **0.832** | **0.808** |
| 2  Snow/Ice to Water | 0.721 | 0.947 |  | |
| 3  Snow/Ice to Barren* Ground | 0.864 | 0.916 |  |  |
| 4  Snow/Ice to Vegetation | 0.565 | 0.929 |  |  |
| 5  Water to Snow/Ice | 0.407 | 1.000 |  |  |
| 6  Water to Water | 0.807 | 0.906 |  |  |
| 7  Water to Barren* Ground | 0.594 | 1.000 |  |  |
| 8  Water to Vegetation | 0.700 | 1.000 |  |  |
| 9  Barren* Ground to Snow/Ice | 0.625 | 0.786 |  |  |
| 10  Barren* Ground to Water | 0.673 | 0.950 |  |  |
| 11  Barren* Ground to Barren* Ground | 0.960 | 0.725 |  |  |
| 12  Barren* Ground to Vegetation | 0.913 | 0.900 |  |  |
| 13  Vegetation to Snow/Ice | 0.667 | 1.000 |  |  |
| 14  Vegetation to Water | 0.533 | 1.000 |  |  |
| 16  Vegetation to Barren* Ground | 0.544 | 1.000 |  |  |
| 17  Vegetation to Vegetation | 0.950 | 0.765 |  |  |
| Table SI 9. Class specific User’s and Producer’s accuracies, with Overall and Kappa accuracies. | | | | |

The accuracy measures in Table SI 9. can be considered as a series of probabilities that are possible to generate from a logistic binomial regression (A. J. Comber 2013). Essentially, overall accuracy, can be estimated from a logistic regression model of a binomial variable indicating where predicted class equals observed class (0 otherwise), and if the result is logit transformed then the returned values provides an estimate of the probability of overall accuracy being equal to 1 (True). In a similar way user’s accuracy can be estimated from a binomial logistic model of reference data (y) against the classified data (x) and producer ’s accuracy from a regression model of classified data (y) against reference data (x).

Any model can be extended to the spatial case using the geographically weighted framework (see A. Comber et al. 2022). These use a moving kernel and compute a series of local models at different locations in the study area, using data under the kernel, but weighted by distance to the kernel centre. Here the spgwr package (Bivand et al. 2017) was used to undertake the spatial analyses of error and a Gaussian kernel was used for the distance weighting.

A series of logistic regressions were undertaken to estimate the spatial distributions of the user and producer errors for each class. A few things to note:

- the GWR analyses were undertaken over a grid of regression points spaced at 5km, covering a 20 km buffer of the sample locations.
- the spatial data were transformed to North America Lambert Conformal Conic projection (see ) so that distances etc. were planar.
- the kernel was manually set to the include nearest 15 % of the data points (i.e. 419 of 2,799 data points) in each local model.
- overall accuracy can be easily done this way but kappa requires a different approach (it does not directly equate to a logistic regression),
- maps for user and producer accuracy for 1 class have been generated (Class 12, Barren Ground to Vegetation) and these explain the per class variation in accuracy.

The results of these are shown in Figures SI 8., SI 9., and SI 10.


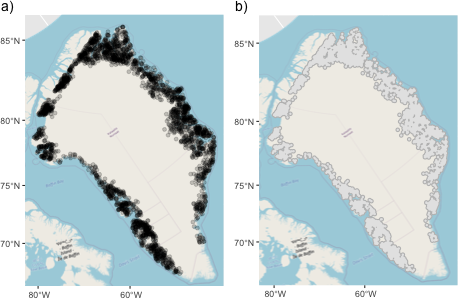


**Figure SI.8**. a) The validation sample locations over a Lambert Conformal Conic projection, and b) the buffered area over which a 5km grid was created for the GW models, with an OpenStreetMap backdrop ⓒ OSM contributors


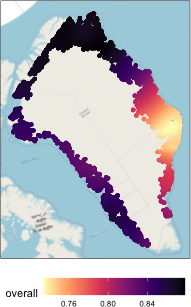


**Figure SI.9.** The GW overall accuracy


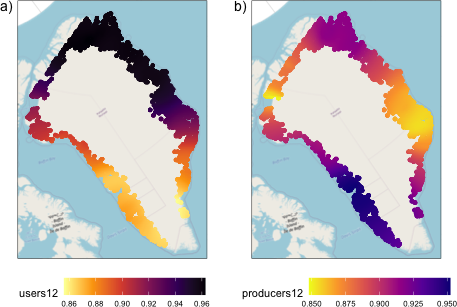


**Figure SI.10**. The GW user (a) and producer (b) accuracies for class 12, barren ground to vegetation

The same geographically weighted accuracy analyses were undertaken for all classes and the results written to a spatial layer (df_gwr_Agg2.gpkg). These are summarised in Table SI 10. and Table SI 11.

|  | Min | Q1 | Median | Mean | Q3 | Max |
| --- | --- | --- | --- | --- | --- | --- |
| users1 | 0.972 | 0.995 | 0.997 | 0.994 | 0.999 | 1.000 |
| users2 | 0.583 | 0.652 | 0.705 | 0.724 | 0.780 | 0.877 |
| users3 | 0.767 | 0.933 | 0.959 | 0.934 | 0.977 | 0.996 |
| users4 | 0.262 | 0.502 | 0.616 | 0.668 | 0.893 | 1.000 |
| users5 | 0.010 | 0.272 | 0.316 | 0.329 | 0.445 | 0.720 |
| users6 | 0.568 | 0.729 | 0.833 | 0.794 | 0.858 | 0.940 |
| users7 | 0.486 | 0.558 | 0.605 | 0.595 | 0.646 | 0.670 |
| users8 | 0.534 | 0.625 | 0.660 | 0.728 | 0.851 | 0.980 |
| users9 | 0.231 | 0.524 | 0.595 | 0.590 | 0.655 | 0.772 |
| users10 | 0.544 | 0.667 | 0.676 | 0.670 | 0.691 | 0.741 |
| users11 | 0.879 | 0.925 | 0.954 | 0.947 | 0.972 | 0.992 |
| users12 | 0.856 | 0.884 | 0.931 | 0.922 | 0.959 | 0.963 |
| users13 | 0.042 | 0.402 | 0.532 | 0.567 | 0.818 | 0.999 |
| users14 | 0.460 | 0.508 | 0.525 | 0.534 | 0.544 | 0.725 |
| users15 | 0.378 | 0.479 | 0.530 | 0.619 | 0.709 | 1.000 |
| users16 | 0.797 | 0.914 | 0.929 | 0.921 | 0.956 | 0.968 |
| Table SI 10. Summaries of the spatial distributions of User accuracies. | | | | | | |

|  | Min | Q1 | Median | Mean | Q3 | Max |
| --- | --- | --- | --- | --- | --- | --- |
| producers1 | 0.449 | 0.547 | 0.682 | 0.671 | 0.783 | 0.895 |
| producers2 | 0.869 | 0.911 | 0.955 | 0.947 | 0.983 | 0.999 |
| producers3 | 0.777 | 0.877 | 0.901 | 0.893 | 0.925 | 0.969 |
| producers4 | 0.833 | 0.920 | 0.996 | 0.960 | 1.000 | 1.000 |
| producers5 | 1.000 | 1.000 | 1.000 | 1.000 | 1.000 | 1.000 |
| producers6 | 0.857 | 0.892 | 0.904 | 0.901 | 0.911 | 0.933 |
| producers7 | 1.000 | 1.000 | 1.000 | 1.000 | 1.000 | 1.000 |
| producers8 | 1.000 | 1.000 | 1.000 | 1.000 | 1.000 | 1.000 |
| producers9 | 0.485 | 0.665 | 0.795 | 0.755 | 0.824 | 0.918 |
| producers10 | 0.850 | 0.921 | 0.964 | 0.944 | 0.977 | 0.987 |
| producers11 | 0.465 | 0.553 | 0.674 | 0.673 | 0.809 | 0.837 |
| producers12 | 0.848 | 0.873 | 0.901 | 0.900 | 0.916 | 0.952 |
| producers13 | 1.000 | 1.000 | 1.000 | 1.000 | 1.000 | 1.000 |
| producers14 | 1.000 | 1.000 | 1.000 | 1.000 | 1.000 | 1.000 |
| producers15 | 1.000 | 1.000 | 1.000 | 1.000 | 1.000 | 1.000 |
| producers16 | 0.557 | 0.708 | 0.740 | 0.746 | 0.791 | 0.842 |
| Table SI 11. Summaries of the spatial distributions of Producer accuracies. | | | | | | |

It is possible to map the user and producer accuracy surfaces for comparison as shown in Figures and SI 11. and SI 12.


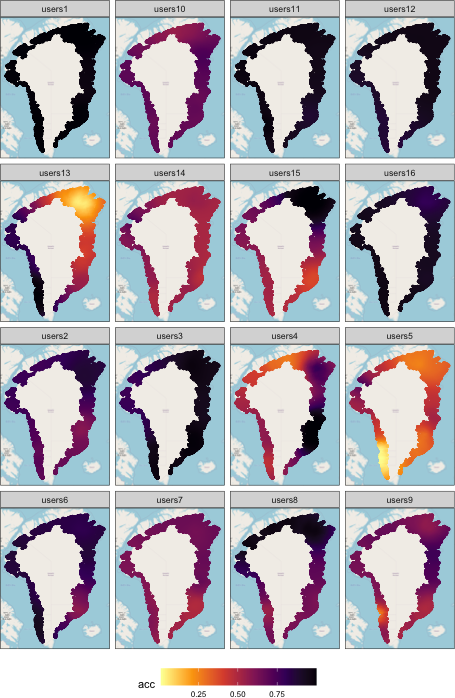


**Figure SI.11**. Mapped GW user accuracies


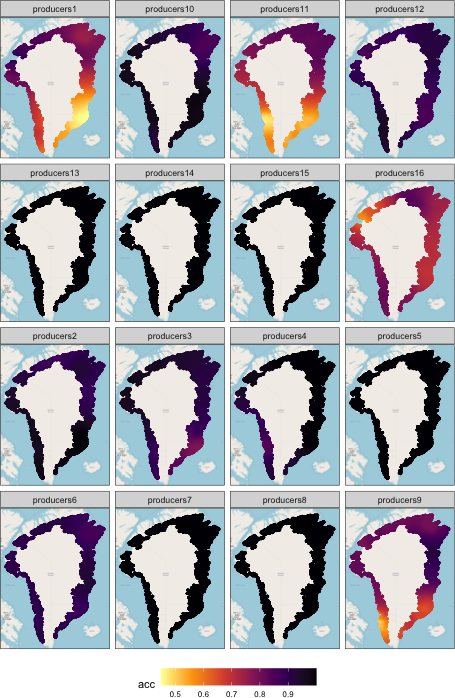


**Figure SI.12**. Mapped GW producer accuracies

1. **Regional patterns of land cover change**

To assess land cover change (LCC) at a regional/national scale across the entirety of Greenland we aggregate our 30 m resolution classifications onto a 10 km x 10 km (100 km^2^) fishnet grid. We were then able to calculate the percentage change in each land cover class within each 100 km^2^ grid cell using the Zonal Statistics as Table tool from the spatial analyst toolbox in ArcMap from ESRI. To ensure that only legitimate land cover changes were measured we masked from both classifications any overlapping cells where one or both had a value of NoData or “Bad Data” prior to calculating percentages.

1. **Local scale process driving change analysis**

Using the regional maps we looked for four locations to highlight the class changes and processes operating at the finest 30 m scale allowed by our data. We identified four key sites for geomorphological process analysis at the native 30 m resolution using the regional patterns of change grids and comparison of the two classifications. Our spatially distributed change accuracy assessment (outlined above) allowed us to select sites in locations where the changes we wished to identify were most accurate. These spatially distributed sites were chosen to highlight four modes of landcover change and the associated geomorphological processes which drive this, therefore exemplifying the utility of the unprecedented 30m resolution classifications underlying the regional change maps. We utilised the same 64 class change image produced for change accuracy assessment outlined above to investigate geomorphological processes and change. We reclassified and aggregated classes from the 64 class change map to highlight geomorphological features and changes, demonstrated in figure 3 of the main manuscript.

**Associations with climate**

Multiple studies have shown that GWR is more accurate and necessary when using climatic predictors for environmental and landscape variables, for both descriptive and predictive usage (e.g. Wang and Tenhunen, 2005; Propastin et al., 2008, and Usman et al. 2013). For a detailed description of GWR see: Brundson et al. 1996, and Fotheringham et al. 2003. Essentially each data point is weighted based on its distance from the kernel cell within the bounds of the bandwidth (neighbourhood). The best GWR model and bandwidth may be determined from a number of model outputs and criteria. In the first order the best bandwidth and model may be determined using the Akaike Information Criterion (AIC). Lower AIC is desirable and generally reflects a closer model approximation to reality, however, involves a trade- off between goodness-of-fit and degrees-of-freedom. Fotheringham et al 2003 define the AIC as:

|  | $AIC= 2n{log}_{e}\left( \hat{\sigma} \right)+n{log}_{e}\left( 2\pi\right)=n\left\{ \frac{n+tr(S)}{n-2-tr(S)} \right\}$ | (4) |
| --- | --- | --- |

where *n* is sample size, $\hat{\sigma}$ is standard deviation of the error term (estimated), and *tr(S)* is the trace of the hat matrix as a function of bandwidth (Fotheringham et al., 2003). R^2^ and adjusted R^2^ are commonly used as indicators model quality so the understanding of the dependant variable based on variation in the independent(s). However, in GWR these will automatically improve towards 1 as bandwidth decreases sufficiently and therefore AIC is a better indicator is it is adjusted for degrees of freedom so is considered here as a better indicator of GWR model quality, though we still publish R^2^ here alongside. Spatial autocorrelation of residuals is also considered when assessing model quality, and so we present Moran’s *I* and associated z-scores. Values closer to 0 of each are more desirable, yet higher values may indicate hot-spots of activity outside the regional norm. Our OLS results therefore supported our use of Geographically Weighted Regression (Table SI.12) OLS and GWR was conducted in ArcMap for each land cover class against the three DDDAT grids at multiple band widths (5 km, 10 km, and 20 km) as well as an AIC determined bandwidth. All OLS results indicate that GWR was necessary. Regression results are shown in Table SI.12.

| Land cover Class (Dependent Variable) | DDDAT Grid | Regression model | R^2^ | Adjusted R^2^ | AIC | Morans *I* autocorrelation of residuals (z-score) |
| --- | --- | --- | --- | --- | --- | --- |
| Snow/Ice | 0^°^C | Global OLS | 0.005 | 0.005 | 50,597.28 | 0.5 (112.8) |
|  |  | GWR bandwidth 50 km | 0.58 | 0.55 | 36,425.37 | 0.18(38.3) |
|  |  | GWR bandwidth 100 km | 0.43 | 0.43 | 37,722.43 | 0.32 (68.3) |
|  |  | GWR bandwidth AIC determined(33 km) | 0.67 | 0.63 | 35,610 | 0.08 (18.7) |
|  | 3^°^C | Global OLS | 0.005 | 0.005 | 40,599.15 | 0.53 (113.7) |
|  |  | GWR bandwidth 50 km | 0.57 | 0.54 | 36,553.13 | 0.21(43.6) |
|  |  | GWR bandwidth 100 km | 0.43 | 0.42 | 37,773.14 | 0.33(70.1) |
|  |  | GWR bandwidth AIC determined (31 km) | 0.67 | 0.63 | 35,630.04 | 0.1 (21.4) |
|  | **6^°^C** | Global OLS | 0.023 | 0.023 | 40,497.39 | 0.52 (111.75) |
|  |  | GWR bandwidth 50 km | 0.58 | 0.55 | 36,398.40 | 0.19(40.5) |
|  |  | GWR bandwidth 100 km | 0.41 | 0.40 | 37,930.84 | 0.34(71.5) |
|  |  | **GWR bandwidth AIC determined (29 km)** | **0.69** | **0.65** | **35,348.05** | **0.06 (13.4)** |
| Meltwater | 0^°^C | Global OLS | 0.000 | 0.000 | 26,663.54 | 0.23(48.7) |
|  |  | GWR bandwidth 50 km | 0.19 | 0.14 | 25,955.45 | 0.10(20.3) |
|  |  | GWR bandwidth 100 km | 0.09 | 0.07 | 26,318.84 | 0.17(35.1) |
|  |  | GWR bandwidth AIC determined(32km) | 0.30 | 0.21 | 25,641.99 | 0.03(7.3) |
|  | 3^°^C | Global OLS | 0.002 | 0.002 | 26,654.54 | 0.23(48.9 |
|  |  | GWR bandwidth 50 km | 0.21 | 0.16 | 25,854.65 | 0.09(18.1) |
|  |  | GWR bandwidth 100 km | 0.10 | 0.08 | 26,239.56 | 0.16(33.4) |
|  |  | GWR bandwidth AIC determined(31km) | 0.32 | 0.24 | 25,524.29 | 0.02(4.7) |
|  | **6^°^C** | Global OLS | 0.000 | 0.000 | 26,663.21 | 0.23(48.5) |
|  |  | GWR bandwidth 50 km | 0.19 | 0.15 | 25,880.75 | 0.09(19.8) |
|  |  | GWR bandwidth 100 km | 0.09 | 0.08 | 26,280.54 | 0.16(34.9) |
|  |  | **GWR bandwidth AIC determined(29km)** | **0.33** | **0.24** | **25,462.50** | **0.02(3.9)** |
| Freshwater | 0^°^C | Global OLS | 0.002 | 0.002 | 22,565.73 | 0.36(77.2) |
|  |  | GWR bandwidth 50 km | 0.38 | 0.34 | 20,453.23 | 0.12(25.3) |
|  |  | GWR bandwidth 100 km | 0.24 | 0.22 | 21,275.31 | 0.20(42.5) |
|  |  | GWR bandwidth AIC determined(32km) | 0.47 | 0.41 | 19,997.55 | 0.05(10.1) |
|  | 3^°^C | Global OLS | 0.003 | 0.003 | 22,560.7 | 0.36(76.6) |
|  |  | GWR bandwidth 50 km | 0.35 | 0.31 | 20,716.15 | 0.14(28.9) |
|  |  | GWR bandwidth 100 km | 0.23 | 0.21 | 21,340.74 | 0.21(44.0) |
|  |  | GWR bandwidth AIC determined(31km) | 0.47 | 0.41 | 20,078.58 | 0.05(11.2) |
|  | **6^°^C** | Global OLS | 0.038 | 0.038 | 22,371.67 | 0.33(69.7) |
|  |  | GWR bandwidth 50 km | 0.34 | 0.31 | 20,736.15 | 0.12(26.4) |
|  |  | GWR bandwidth 100 km | 0.21 | 0.2 | 21,405.21 | 0.2(42.9) |
|  |  | **GWR bandwidth AIC determined(29km)** | **0.49** | **0.42** | **19,943.43** | **0.03(6.6)** |
| Coarse Sediment | 0^°^C | Global OLS | 0.002 | 0.002 | 36,584.89 | 0.56(118.4) |
|  |  | GWR bandwidth 50 km | 0.49 | 0.46 | 33,393.42 | 0.20(42.7) |
|  |  | GWR bandwidth 100 km | 0.35 | 0.34 | 34,400.79 | 0.34(71.7) |
|  |  | GWR bandwidth AIC determined(32km) | 0.59 | 0.55 | 32,638.55 | 0.11(22.6) |
|  | 3^°^C | Global OLS | 0.007 | 0.007 | 36,559.23 | 0.56(117.5) |
|  |  | GWR bandwidth 50 km | 0.51 | 0.48 | 33,240.38 | 0.19(40.8) |
|  |  | GWR bandwidth 100 km | 0.37 | 0.36 | 34,245.79 | 0.33(68.8) |
|  |  | GWR bandwidth AIC determined(31km) | 0.61 | 0.57 | 32,414.13 | 0.09(19.1) |
|  | **6^°^C** | Global OLS | 0.005 | 0.005 | 36,570.56 | 0.56(118.5) |
|  |  | GWR bandwidth 50 km | 0.50 | 0.48 | 33,248.29 | 0.20(41.6) |
|  |  | GWR bandwidth 100 km | 0.37 | 0.36 | 34,286.52 | 0.34(71.0) |
|  |  | **GWR bandwidth AIC determined(29km)** | **0.62** | **0.57** | **32,335.74** | **0.07(15.8)** |
| Fine-grained Sediment | 0^°^C | Global OLS | 0.008 | 0.008 | 33,198.79 | 0.45(94.6) |
|  |  | GWR bandwidth 50 km | 0.35 | 0.32 | 31,337.99 | 0.18(39.0) |
|  |  | GWR bandwidth 100 km | 0.21 | 0.20 | 32,114.55 | 0.29(60.8) |
|  |  | GWR bandwidth AIC determined(32km) | 0.47 | 0.41 | 30,728.22 | 0.09(20.9) |
|  | 3^°^C | Global OLS | 0.003 | 0.002 | 33,227.9 | 0.45(95.9) |
|  |  | GWR bandwidth 50 km | 0.35 | 0.31 | 31,358.15 | 0.19(40.0) |
|  |  | GWR bandwidth 100 km | 0.20 | 0.19 | 32,192.42 | 0.30(63.1) |
|  |  | GWR bandwidth AIC determined(31km) | 0.48 | 0.41 | 30,695.59 | 0.09(19.7) |
|  | **6^°^C** | Global OLS | 0.005 | 0.005 | 33,213.46 | 0.45(95.5) |
|  |  | GWR bandwidth 50 km | 0.35 | 0.31 | 31,388.73 | 0.19(40.4) |
|  |  | GWR bandwidth 100 km | 0.21 | 0.19 | 32,513.16 | 0.30(62.9) |
|  |  | **GWR bandwidth AIC determined(29km)** | **0.50** | **0.43** | **30,510.02** | **0.07(15.7)** |
| Bedrock | 0^°^C | Global OLS | 0.003 | 0.003 | 40,722.53 | 0.56(118.9) |
|  |  | GWR bandwidth 50 km | 0.58 | 0.55 | 36,548.43 | 0.18(38.4) |
|  |  | GWR bandwidth 100 km | 0.44 | 0.43 | 37,802.57 | 0.33(70.0) |
|  |  | GWR bandwidth AIC determined(32km) | 0.66 | 0.62 | 35,832.20 | 0.08(17.9) |
|  | 3^°^C | Global OLS | 0.000 | 0.000 | 40,738.89 | 0.57(119.8) |
|  |  | GWR bandwidth 50 km | 0.58 | 0.56 | 36,462.45 | 0.18(38.9) |
|  |  | GWR bandwidth 100 km | 0.45 | 0.44 | 37,690.69 | 0.32(69.4) |
|  |  | GWR bandwidth AIC determined(31km) | 0.68 | 0.64 | 35,610.95 | 0.07(15.8) |
|  | **6^°^C** | Global OLS | 0.000 | 0.000 | 40,737.03 | 0.57(119.9) |
|  |  | GWR bandwidth 50 km | 0.58 | 0.56 | 36,459.76 | 0.17(36.7) |
|  |  | GWR bandwidth 100 km | 0.43 | 0.42 | 37,866.89 | 0.34(70.9) |
|  |  | **GWR bandwidth AIC determined(29km)** | **0.69** | **0.65** | **35,585.63** | **0.05(11.4)** |
| Dry Tundra | 0^°^C | Global OLS | 0.002 | 0.002 | 37,989.99 | 0.59(124.1) |
|  |  | GWR bandwidth 50 km | 0.50 | 0.47 | 34,710.29 | 0.28(59.9) |
|  |  | GWR bandwidth 100 km | 0.28 | 0.27 | 36,386.68 | 0.45(94.9) |
|  |  | GWR bandwidth AIC determined(32km) | 0.62 | 0.58 | 33,619.47 | 0.16(33.2) |
|  | 3^°^C | Global OLS | 0.003 | 0.003 | 37,983.55 | 0.59(123.9) |
|  |  | GWR bandwidth 50 km | 0.49 | 0.46 | 34,833.01 | 0.29(62.5) |
|  |  | GWR bandwidth 100 km | 0.27 | 0.26 | 36,432.33 | 0.45(96.7) |
|  |  | GWR bandwidth AIC determined(31km) | 0.64 | 0.60 | 33,497.23 | 0.15(32.2) |
|  | **6^°^C** | Global OLS | 0.017 | 0.017 | 37,909.40 | 0.58(122.9) |
|  |  | GWR bandwidth 50 km | 0.52 | 0.50 | 34,439.45 | 0.26(55.6) |
|  |  | GWR bandwidth 100 km | 0.29 | 0.27 | 36,296.88 | 0.45(94.5) |
|  |  | **GWR bandwidth AIC determined(29km)** | **0.67** | **0.63** | **33,087.02** | **0.11(22.6)** |
| Dense/wet Vegetation | 0^°^C | Global OLS | 0.001 | 0.001 | 29,311.42 | 0.57(121.6) |
|  |  | GWR bandwidth 50 km | 0.49 | 0.46 | 26,113.14 | 0.22(46.8) |
|  |  | GWR bandwidth 100 km | 0.34 | 0.33 | 27,265.46 | 0.36(75.8) |
|  |  | GWR bandwidth AIC determined(32km) | 0.60 | 0.55 | 25,253.33 | 0.11(23.6) |
|  | 3^°^C | Global OLS | 0.017 | 0.017 | 29,226.08 | 0.56(118.4) |
|  |  | GWR bandwidth 50 km | 0.50 | 0.47 | 26,047.58 | 0.22(45.6) |
|  |  | GWR bandwidth 100 km | 0.34 | 0.33 | 27,204.55 | 0.35(75.1) |
|  |  | GWR bandwidth AIC determined(31km) | 0.61 | 0.57 | 25,168.41 | 0.09(20.1) |
|  | **6^°^C** | Global OLS | 0.058 | 0.058 | 28,998.04 | 0.54(113.8) |
|  |  | GWR bandwidth 50 km | 0.50 | 0.47 | 26,005.77 | 0.21(44.9) |
|  |  | GWR bandwidth 100 km | 0.35 | 0.34 | 27,165.78 | 0.35(73.9) |
|  |  | **GWR bandwidth AIC determined(29km)** | **0.63** | **0.58** | **25,031.95** | **0.07(16.9)** |
| Table SI 12. Ordinary Least Squares (OLS) and Geographically Weighted Regression (GWR) results for every class against the three Difference in Degree Day Above Temperature Grids (DDDAT). Bold and underlined values represent best (highest R^2^ and lowest AIC) regression results per class and DDDAT. | | | | | | |

1. **Landcover Change Phase Model of Greenland**

To produce a model of landcover phase changes across Greenland we utilised the 64 class change image as referenced previously (Table SI.5). This was dissected into 20 latitudinal bands covering 2 degrees of latitude each, 10 bands for the east and west (see Figure 4 of main text). For each of these latitudinal bands an 8x8 matrix was produced of class transitions, with each cell of the matrix showing cell counts for each transition, and the diagonals being static, i.e. no change. These cell counts were then converted into area (km^2^) and percentage of each matrix, both considering and exclusing static landcover cells (Figure SI 6 &Figure SI 7). These matrices were then colour coded by proportion of that matrix each cell represents, and then again but comparing that transition cell against the same transition cell in each of the other matrices. In short, the predominant inter-class land cover changes were defined per latitudinal band and separately for the east and west coasts.

**Figure SI.13.** Matrices of class transition. Cell values represent percentage of latitudinal band area covered by this transition. Static surfaces in the diagonals are removed and not considered in percentages as we are only concerned with defining class transitions here. Numbers on the right represent latitudinal band (degrees) and the columns denote coast (i.e. left: west, right: east). Colour coding is per-matrix, i.e. colour scaled from red (low occurrence) to green (high occurrence) within that latitudinal band.

**Figure SI.14.** Matrices of class transition where cell values represent percentage of latitudinal band area covered by this transition. Static surfaces in the diagonals are removed and not considered in percentages as we are only concerned with defining class transitions here. Numbers on the right represent latitudinal band (degrees) and the columns denote coast (i.e. left: west, right: east). Colour coding is relative to that cell transition in each of the other matrixes, i.e. colour scaled from red (low occurrence) to green (high occurrence) for that exact transition across all matrices.

1. **References**
2. Bannari, A., Morin, D., Bonn, F. & Huete, A. R. 1995. A review of vegetation indices. Remote Sensing Reviews, 13, 95-120.
3. Bivand, Roger, Danlin Yu, Tomoki Nakaya, Miquel-Angel Garcia-Lopez, and Maintainer Roger Bivand. 2017. “Package ‘Spgwr’.” R Software Package.
4. Breiman, L., 2001. Random forests. Machine learning, 45(1), pp.5-32.
5. Brunsdon, C., Fotheringham, A.S. and Charlton, M.E., 1996. Geographically weighted regression: a method for exploring spatial nonstationarity. Geographical analysis, 28(4), pp.281-298.
6. Carlson, T. N. & Ripley, D. A. 1997. On the relation between NDVI, fractional vegetation cover, and leaf area index. Remote Sensing of Environment, 62, 241-252.
7. Carrivick, J. L., Yde, J., Russell, A. J., Quincey, D. J., Ingeman-Nielsen, T. & Mallalieu, J. 2017. Ice-margin and meltwater dynamics during the mid-Holocene in the Kangerlussuaq area of west Greenland. Boreas, 46, 369-387.
8. Clifford, P., Richardson, S. and Hemon, D., 1989. Assessing the significance of the correlation between two spatial processes. Biometrics, pp.123-134.
9. Cohen, W. B. & Goward, S. N. 2004. Landsat's role in ecological applications of remote sensing. Bioscience, 54, 535-545.
10. Comber, Alexis J. 2013. “Geographically Weighted Methods for Estimating Local Surfaces of Overall, User and Producer Accuracies.” Remote Sensing Letters 4 (4): 373–80.
11. Comber, Alexis, Chris Brunsdon, Martin Callaghan, Paul Harris, Binbin Lu, and Nick Malleson. 2022. “Gwverse: A Template for a New Generic Geographically Weighted Rpackage.” Geographical Analysis https://doi.org/10.1111/gean.12337.
12. Comber, Alexis, Chris Brunsdon, Martin Charlton, and Paul Harris. 2017. “Geographically Weighted Correspondence Matrices for Local Error Reporting and Change Analyses: Mapping the Spatial Distribution of Errors and Change.” Remote Sensing Letters 8 (3): 234–43.
13. Comber, Alexis, Peter Fisher, Chris Brunsdon, and Abdulhakim Khmag. 2012. “Spatial Analysis of Remote Sensing Image Classification Accuracy.” Remote Sensing of Environment 127: 237–46.
14. Tucker, C.J., 1979. Red and photographic infrared linear combinations for monitoring vegetation. Remote sensing of Environment, 8(2), pp.127-150.
15. Congalton, Russell G. 1991. “A Review of Assessing the Accuracy of Classifications of Remotely Sensed Data.” Remote Sensing of Environment 37 (1): 35–46.
16. Díaz-Delgado, R., Bustamante, J., Aragonés, D. and Pacios, F., 2006. Determining water body characteristics of Doñana shallow marshes through remote sensing. Institute of Electrical and Electronics Engineers.
17. Foody, G.M., 2004. Thematic map comparison. Photogrammetric Engineering & Remote Sensing, 70(5), pp.627-633.
18. Foody, GM. 2005. “Local Characterization of Thematic Classification Accuracy Through Spatially Constrained Confusion Matrices.” International Journal of Remote Sensing 26 (6): 1217–28.
19. Fotheringham, A.S., Brunsdon, C. and Charlton, M., 2003. Geographically weighted regression: the analysis of spatially varying relationships. John Wiley & Sons.
20. Gorelick, N., Hancher, M., Dixon, M., Ilyushchenko, S., Thau, D. & Moore, R. 2017. Google Earth Engine: Planetary-scale geospatial analysis for everyone. Remote Sensing of Environment, 202, 18-27.
21. Goward, S., Arvidson, T., Williams, D., Faundeen, J., Irons, J. and Franks, S., 2006. Historical record of Landsat global coverage. Photogrammetric Engineering & Remote Sensing, 72(10), pp.1155-1169.
22. Hall, D. K. & Riggs, G. A. 2011. Normalized-Difference Snow Index (NDSI). In: Singh, V. P., Singh, P. & Haritashya, U. K. (eds.) Encyclopedia of Snow, Ice and Glaciers. Dordrecht: Springer Netherlands.
23. Hasmadi, M., Pakhriazad, H.Z. and Shahrin, M.F., 2009. Evaluating supervised and unsu-pervised techniques for land cover mapping using remote sensing data. Geografia: Malaysian Journal of Society and Space, 5(1), pp.1-10.
24. Howat, I. M., Negrete, A. & Smith, B. E. 2014. The Greenland Ice Mapping Project (GIMP) land classification and surface elevation data sets. Cryosphere, 8, 1509-1518.
25. Hurni, K., Heinimann, A., & Würsch, L., 2017. Google Earth Engine Image Pre-processing Tool: Background and Methods. Available from: https://www.cde.unibe.ch/e65013/e542846/e707304/e707386/e707390/CDE_Pre-processingTool-UserGuide_eng.pdf . Date: 09/12/2021.
26. Jørgensen, C.J., Johansen, K.M.L., Westergaard-Nielsen, A. and Elberling, B., 2015. Net regional methane sink in High Arctic soils of northeast Greenland. Nature Geoscience, 8(1), pp.20-23.
27. Joshi, P. K., Ghosh, A., Chakraborty, A., Sharma, R. & Joshi, A. 2013. Landsat again - continuing remote sensing, monitoring, mapping and measuring. Current Science, 105, 761-763.
28. Karami, M., Westergaard-Nielsen, A., Normand, S., Treier, U.A., Elberling, B. and Hansen, B.U., 2018. A phenology-based approach to the classification of Arctic tundra ecosystems in Greenland. ISPRS journal of photogrammetry and remote sensing, 146, pp.518-529.
29. Kloiber, S. N., Brezonik, P. L., Olmanson, L. G. & Bauer, M. E. 2002. A procedure for regional lake water clarity assessment using Landsat multispectral data. Remote Sensing of Environment, 82, 38-47.
30. Kulkarni, A. V., Mathur, P., Rathore, B. P., Alex, S., Thakur, N. & Kumar, M. 2002. Effect of global warming on snow ablation pattern in the Himalaya. Current Science, 83, 120-123.
31. Li, S., Zhao, Z., Miaomiao, X. and Wang, Y., 2010. Investigating spatial non-stationary and scale-dependent relationships between urban surface temperature and environmental factors using geographically weighted regression. Environmental Modelling & Software, 25(12), pp.1789-1800.
32. Marshall, G.J., Dowdeswell, J.A. and Rees, W.G., 1994. The spatial and temporal effect of cloud cover on the acquisition of high quality Landsat imagery in the European Arctic sector. Remote Sensing of Environment, 50(2), pp.149-160.
33. Mcfeeters, S. K. 1996. The use of the normalized difference water index (NDWI) in the delineation of open water features. International Journal of Remote Sensing, 17, 1425-1432.
34. Nolin, A. W. 2010. Recent advances in remote sensing of seasonal snow. Journal of Glaciology, 56, 1141-1150.
35. F0sson, P., Foody, G.M., Stehman, S.V. and Woodcock, C.E., 2013. Making better use of accuracy data in land change studies: Estimating accuracy and area and quantifying uncertainty using stratified estimation. Remote Sensing of Environment, 129, pp.122-131.
36. Pontius Jr, R.G. and Millones, M., 2011. Death to Kappa: birth of quantity disagreement and allocation disagreement for accuracy assessment. International Journal of Remote Sensing, 32(15), pp.4407-4429.
37. Pontius Jr, Robert Gilmore, and Alı́ Santacruz. 2014. “Quantity, Exchange, and Shift Components of Difference in a Square Contingency Table.” International Journal of Remote Sensing 35 (21): 7543–54.
38. Pontius Jr, Robert Gilmore, and Marco Millones. 2011. “Death to Kappa: Birth of Quantity Disagreement and Allocation Disagreement for Accuracy Assessment.” International Journal of Remote Sensing 32 (15): 4407–29.
39. Poortinga, A., Tenneson, K., Shapiro, A., Nquyen, Q., San Aung, K., Chishtie, F. & Saah, D. 2019. Mapping Plantations in Myanmar by Fusing Landsat-8, Sentinel-2 and Sentinel-1 Data along with Systematic Error Quantification. Remote Sensing, 11
40. Propastin, P., Kappas, M. and Erasmi, S., 2008. Application of geographically weighted regression to investigate the impact of scale on prediction uncertainty by modelling relationship between vegetation and climate. International journal of spatial data infrastructures research, 3(3), pp.73-94.
41. Rodriguez-Galiano, V. F., Ghimire, B., Rogan, J., Chica-Olmo, M. & Rigol-Sanchez, J. P. 2012b. An assessment of the effectiveness of a random forest classifier for land-cover classification. Isprs Journal of Photogrammetry and Remote Sensing, 67, 93-104.
42. Selkowitz, D. & Forster, R. 2015. An Automated Approach for Mapping Persistent Ice and Snow Cover over High Latitude Regions. Remote Sensing, 8.
43. Soenen, S. A., Peddle, D. R. & Coburn, C. A. 2005. SCS+C: A modified sun-canopy-sensor topographic correction in forested terrain. Ieee Transactions on Geoscience and Remote Sensing, 43, 2148-2159.
44. Tømmervik, H., Høgda, K.A. and Solheim, I., 2003. Monitoring vegetation changes in Pasvik (Norway) and Pechenga in Kola Peninsula (Russia) using multitemporal Landsat MSS/TM data. Remote sensing of Environment, 85(3), pp.370-388.
45. Tucker, C. J., Grant, D. M. & Dykstra, J. D. 2004. NASA's global orthorectified landsat da-ta set. Photogrammetric Engineering and Remote Sensing, 70, 313-322.
46. Usgs. 2019. Landsat Surface Reflectance [Online]. Available: https://www.usgs.gov/land-resources/nli/landsat/landsat-surface-reflectance?qt-science_support_page_related_con=1#qt-science_support_page_related_con [Accessed 29 July 2021]
47. Usman, U., Yelwa, S.A., Gulumbe, S.U., Danbaba, A. and Nir, R., 2013. Modelling relationship between NDVI and climatic variables using geographically weighted regression. Journal of Mathematical Sciences and Applications, 1(2), pp.24-28.
48. Vanonckelen, S., Lhermitte, S. & Van Rompaey, A. 2013. The effect of atmospheric and topographic correction methods on land cover classification accuracy. International Journal of Applied Earth Observation and Geoinformation, 24, 9-21.
49. Wang, J., Price, K. P. and P. M. Rich. (2001). Spatial patterns of NDVI in response to precipitation and temperature in the central Great Plains. International Journal of Remote Sensing, 22: 3827-3844.
50. Wang, Q., Ni, J. and Tenhunen, J., 2005. Application of a geographically‐weighted regression analysis to estimate net primary production of Chinese forest ecosystems. Global ecology and biogeography, 14(4), pp.379-393.
51. Wulder, M.A., White, J.C., Goward, S.N., Masek, J.G., Irons, J.R., Herold, M., Cohen, W.B., Loveland, T.R. and Woodcock, C.E., 2008. Landsat continuity: Issues and opportunities for land cover monitoring. Remote Sensing of Environment, 112(3), pp.955-969.
52. Xu, H. 2006. Modification of normalised difference water index (NDWI) to enhance open water features in remotely sensed imagery. International Journal of Remote Sensing, 27, 3025-3033.
53. Zhao, N., Yang, Y. and Zhou, X., 2010. Application of geographically weighted regression in estimating the effect of climate and site conditions on vegetation distribution in Haihe Catchment, China. Plant Ecology, 209(2), pp.349-359.
54. Zhou, H., Wang, J.A., Wan, J. and Jia, H., 2010. Resilience to natural hazards: a geographic perspective. Natural hazards, 53(1), pp.21-41.
